# Band-collision gel electrophoresis

**DOI:** 10.1038/s41467-019-11438-9

**Published:** 2019-08-12

**Authors:** Dimitri A. Bikos, Thomas G. Mason

**Affiliations:** 1 Department of Chemistry and Biochemistry, University of California, Los Angeles, Los Angeles, CA 90095 USA; 2 Department of Physics and Astronomy, University of California, Los Angeles, Los Angeles, CA 90095 USA

**Keywords:** Chemical engineering, Chemical physics, Imaging techniques, Biochemical assays, Reaction kinetics and dynamics

## Abstract

Electrophoretic mobility shift assays are widely used in gel electrophoresis to study binding interactions between different molecular species loaded into the same well. However, shift assays can access only a subset of reaction possibilities that could be otherwise seen if separate bands of reagent species might instead be collisionally reacted. Here, we adapt gel electrophoresis by fabricating two or more wells in the same lane, loading these wells with different reagent species, and applying an electric field, thereby producing collisional reactions between propagating pulse-like bands of these species, which we image optically. For certain pairs of anionic and cationic dyes, propagating bands pass through each other unperturbed; yet, for other pairs, we observe complexing and precipitation reactions, indicating strong attractive interactions. We generalize this band-collision gel electrophoresis (BCGE) approach to other reaction types, including acid-base, ligand exchange, and redox, as well as to colloidal species in passivated large-pore gels.

## Abstract (web summary)

Electrophoretic mobility shift assays are widely used in gel electrophoresis to study binding interactions between different molecular species, but these assays access only a subset of reaction possibilities. Here, the authors develop a band-collision gel electrophoresis (BCGE) approach that demonstrates a much wider variety of reaction types.

## Introduction

Gel electrophoresis (GE) is a powerful technique for characterizing and separating solvated ionic molecular or colloidal species based on their electrophoretic mobilities *μ*_e_. These species are loaded into wells in a nanoporous elastic gel immersed in an electrolyte buffer solution^1–6^, and an electric field is then applied between two inert electrodes. For analyzing poly-anionic DNA and RNA, GE is an extremely important technology, providing high-resolution measurements of lengths of poly-nucleic acids^7–9^. Moreover, through sodium dodecyl sulfate–polyacrylamide gel electrophoresis (SDS-PAGE)^10–12^, GE can be used with a wide range of proteins. Pulsed^13–15^, 2D^16–18^, and 3D^19–21^ forms have further broadened GE.

Electrophoretic mobility shift assays (EMSAs)^22–24^ have extended GE significantly by probing interactions between macromolecular species, such as proteins and poly-nucleic acids, loaded into the same single well in a gel. After a pre-specified duration of interaction, an electric field is applied to induce separation. EMSAs are typically used to probe equilibrium biomolecular binding, since two bound species would typically propagate differently than either separately, and is therefore important in the field of genomic expression^25,26^. Beyond equilibrium binding, EMSAs have also been used to measure dissociation kinetics^27–35^. In addition, relative binding constants between competing ligands, orders of reactions, rate constants, and Arrhenius parameters can be measured using EMSAs^28^. In some cases, EMSAs have also assisted in deducing reaction mechanisms^30^ and the presence of reactive intermediates^33^. Variability in binding efficiencies between certain mutant and wild-type enzymes have been discerned by EMSAs^27^. Certain short-lived transient protein–DNA complexes can persist for hours in a polyacrylamide gel matrix during EMSAs^31^; this persistence likely arises from cage effects caused by the gel matrix that strongly reduce the rate of decomplexing of such long biomolecules^29^. Thus, EMSAs have been used to study kinetics not only in solution (e.g. within loading wells) but also within the porous gel matrix. EMSAs have also been performed at different pH and ionic strength^34^. While EMSAs have provided useful insights into biomolecular binding, loading reactant species into the same well limits the use of the protocol inherent to EMSAs for other types of reactions. Correspondingly, a gel dead-time limits the temporal resolution of kinetics, since after activating the electric field it is typically necessary to wait for any complexes that might have been formed to leave the well and enter the gel before such binding interactions can be monitored^28^. Moreover, reaction kinetics could potentially be explored and visualized using a different GE approach that overcomes certain limitations of EMSAs.

Dye molecules would offer many advantages for initially demonstrating such a different GE approach, primarily because many dyes are charged, are typically much smaller than the characteristic pore sizes of gels, and can be readily seen as a consequence of optical absorption. Certain pairs of different dye molecules are known to attract and to form complexes or even precipitates in mixtures of bulk aqueous solutions, as observed decades ago using spectrophotometry^36,37^. The degree of attraction between two different dye molecules can involve electrostatic^38^, hydrophobic^39^, and pi-stacking interactions;^38^ steric effects^40^ and internal flexibility of the molecules can also be important. Moreover, short-range screened electrostatic attractions between acidic (anionic) dyes and basic (cationic) dyes^41^ can enhance complex formation and precipitation, leading to non-additivity in optical absorption spectra of many anionic–cationic dye mixtures. Thus, dye molecules represent an important subset of potential reagents for readily demonstrating any new reactive GE method that goes beyond EMSAs.

**Fig. 1:**
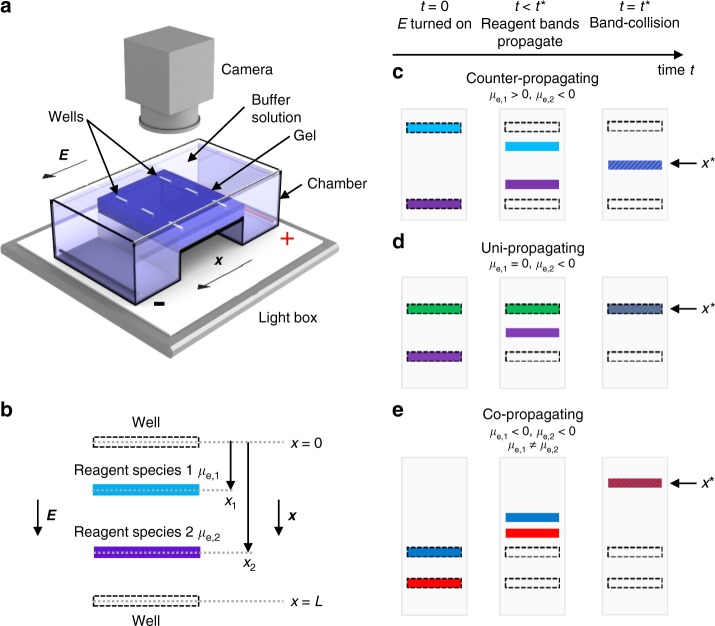
Visualizing reactions between two different optically absorbing reagent species using band-collision gel electrophoresis (BCGE). **a** A gel having two wells per lane is cast and transferred into a transparent horizontal gel electrophoresis chamber filled with a buffer solution at a desired pH. Pt-wires near the ends of the chamber, designated by black (−) and red ( + ), are connected to a power supply (not shown). Each well is loaded with a different reagent species, and the power supply, which generates an electric field *E* that lies along the *x*-direction, is turned on at time *t* = 0. A light box underneath the chamber provides uniform transmission illumination of white visible light, and time-lapse images are captured by an overhead camera with a lens selected to minimize spatial distortion. **b** Overhead view depicting the center locations *x*_1_(*t*) and *x*_2_(*t*) of propagating bands of reagent species 1 (blue band) and 2 (purple band), respectively, in a lane at time *t*; both *x*_1_ and *x*_2_ are referenced relative to the well centered at *x* = 0. Reagent species 1 and 2 were initially loaded into the wells centered at *x* = 0 and *x* = *L*, respectively. Here, the electrophoretic mobilities of reagent species 1 and 2 have *μ*_e,1_ > 0 and *μ*_e,2_ < 0, respectively. **c**–**e** Each panel shows the evolution of bands in a single lane containing two wells during BCGE for different *μ*_e,1_ and *μ*_e,2_ (see the Methods section): (left) at time *t* = 0 when *E* is turned on, (middle) at some time later *t* < t* before band collision, and (right) full band collision at *t* = t*, yielding band-collision location x*. **c** Counter-propagating BCGE: *μ*_e,1_ > 0 and *μ*_e,2 _< 0, so band collision always occurs at x* between the wells. **d** Uni-propagating BCGE: *μ*_e,1_ = 0 (as shown) or *μ*_e,2_ = 0, so the band of charged reagent species collides with the stationary band of uncharged reagent species in its well. **e** Co-propagating BCGE: both *μ*_e,1_ and *μ*_e,2_ have the same sign (<0 as shown) but *μ*_e,1_ ≠ *μ*_e,2_, so band collision occurs at x* outside the region between the wells

Here, we modify traditional GE to control and study the evolution of collisional reactions between two or more reagent species in solution that have different *μ*_e_. We design and fabricate two or more wells in the same lane of the gel, load each of these wells with one or more reagent species, and then apply an electric field. To facilitate optical visualization, we first use anionic and cationic organic dye molecules^42^, some of which have been previously studied individually in agarose^43^ and polyacrylamide^44^ gels, as reagent species. We record high-resolution time-lapse videos of collisions between pulse-like bands of these species (see Fig. 1 and the Methods section), thereby revealing both attractive interactions and also, more generally with other reagent types, irreversible chemical reactions. We extend this approach, which we call band-collision gel electrophoresis (BCGE), to include invisible (i.e., optically non-absorbing or only very weakly absorbing) molecules, as well as colloidal species that scatter visible light such as polymer nanospheres^45^. We show that a wide range of complex spatiotemporal patterns form and evolve when bands of different species collide. Moreover, we show that BCGE can be used to study not only associative intermolecular interactions, such as complexing and precipitation, but also acid–base, redox, and ligand-exchange reactions. Thus, collisional reactions probed using BCGE go well beyond prior investigations reporting propagation of bands of dye molecules without contact in separate lanes. In addition, using BCGE, sequences of reactions can be effectively programmed by appropriately choosing the location of the wells in the same lane and the types of species that are loaded into each of these wells. Thus, BCGE can provide the electrophoretic equivalent of microfluidic manipulation^46–48^ of pulses of solvated and typically charged reagent species, which is reminiscent of pulses of atomic and molecular ions that can be reacted through collisions in vacuo^49–52^.

## Results

### Initial considerations before conducting BCGE experiments

**Fig. 2:**
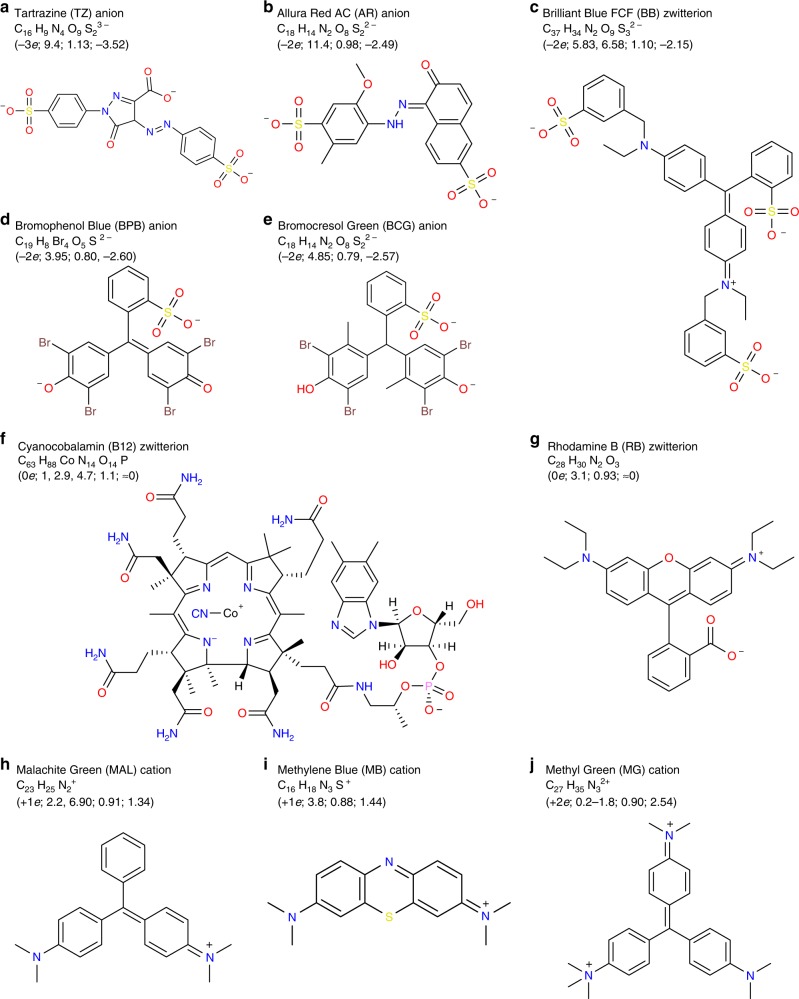
Structural diagrams and properties of organic dyes at pH = 9. **a** Tartrazine (TZ) anion, 465.39 g mol^−1^. **b** Allura Red (AR) anion, 450.44 g mol^−1^. **c** Brilliant Blue FCF (BB) zwitterion, 746.87 g mol^−1^. **d** Bromophenol Blue (BPB) anion, 667.95 g mol^−1^. **e** Bromocresol Green (BCG) anion, 698.02 g mol^−1^. **f** Cyanocobalamin FCF (B12) zwitterion, 1,355.39 g mol^−1^. **g** Rhodamine B (RB) zwitterion, 442.56 g mol^−1^. **h** Malachite Green (MAL) cation, 329.47 g mol^−1^. **i** Methylene Blue (MB) cation, 284.40 g mol^−1^. **j** Methyl Green (MG) cation, 387.57 g mol^−1^. Values in parentheses at the upper left of each panel provide predicted nearest integer charge *q* (*e*); translational hydrodynamic radius *a* (in nm); and measured electrophoretic mobility *μ*_e,meas_ (in 10^−8^ m^2^ V^−1^ s^−1^). Below this, we list reported p*K*_a_ value(s) or range(s) from literature sources (see Supplementary Methods). Charges are approximate, and have been rounded to the nearest integer (see Supplementary Methods). Estimates of equivalent hydrodynamic sphere radii are made using WinHydroPro and HyperChem (see the Methods section). Corresponding minimized molecular models from HyperChem are shown in Supplementary Fig. 1

To facilitate subsequent BCGE experiments, we first measure *μ*_e_ of organic dye molecules in agarose gels, and compare these *μ*_e_ to mobilities predicted by the Smoluchowski equation^53^ and molecular models (see the Methods section, Fig. 2; Supplementary Methods, and Supplementary Fig. 1). Knowing these *μ*_e_, we then design and cast agarose gels having two or more wells in each lane, and we systematically explore complex spatiotemporal patterns that can form when counter-propagating bands of dye molecules collide (see examples in Supplementary Movie 1). We demonstrate this first for complexing and precipitation reactions, summarizing the movies using space–time plots. We also show examples of acid–base, ligand-exchange, and redox reactions, and we generalize the reagents to include colloidal nanoparticles.

### Electrophoretic mobilities of dye molecules

**Fig. 3:**
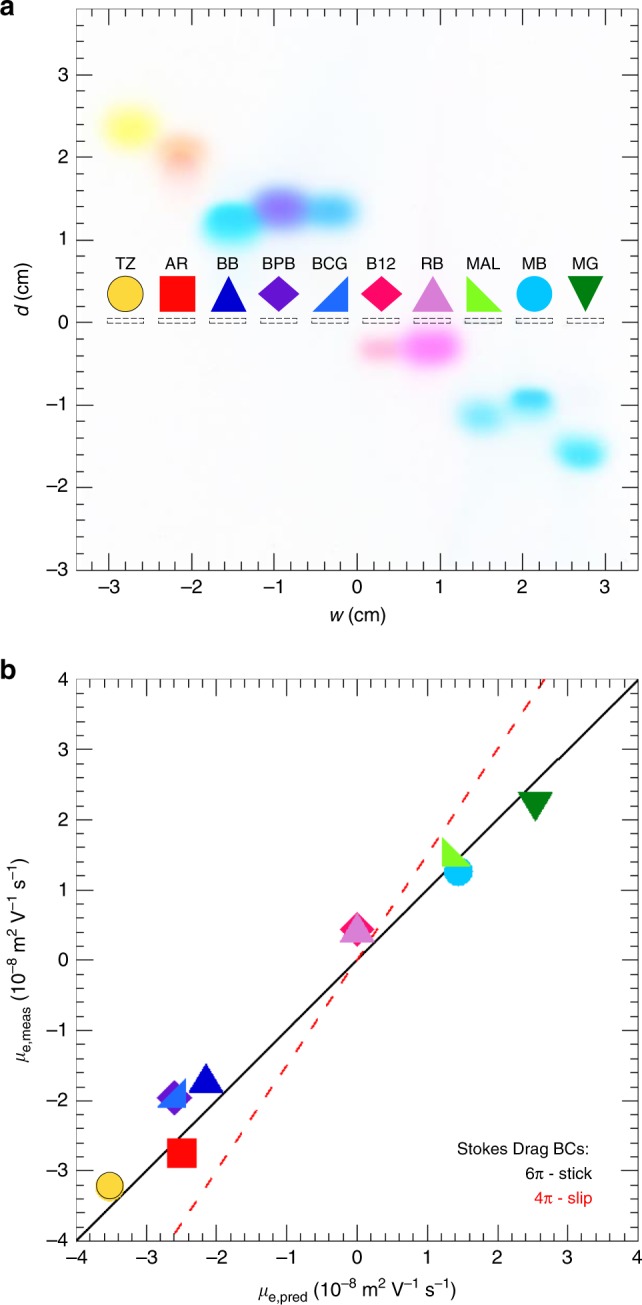
Measured and predicted electrophoretic mobilities of organic dyes. Conditions: 3.0% (w/w) agarose gels; 5.0 mM SBB at pH 9.0; applied electric field *E* = 3.1 V cm^−1^. **a** Image of bands of dye molecules after a propagation time *t* = 2,400 s from wells (dashed rectangles at *d* = 0). Propagation distance along the applied field is *d* and the *x*-direction points downward; transverse distance is designated by *w*. Dyes are identified by abbreviations (see Fig. 2) and assigned symbols. **b** *μ*_e,meas_ versus predicted *μ*_e,pred_ using the Smoluchowski equation with stick boundary conditions for Stokes drag and effective hydrodynamic radii from molecular modeling (points). Black solid line: *μ*_e,meas_ = *μ*_e,pred_ (stick) has ideal slope = 1. Source data are provided as a Source Data file

Using agarose GE in 5.0 mM sodium borate buffer (SBB) at pH = 9.0 at an electric field of *E* = 3.1 V cm^−1^ in the linear propagation regime (see Methods), we measure average velocities *v* of a set of dye molecules (Fig. 2) based on observed propagation distances of bands (Fig. 3a; Supplementary Movie 2), from which we determine *μ*_e,meas_ = *v*/*E*. Moreover, in Fig. 3b, we compare these *μ*_e,meas_ to predictions given by the Smoluchowski equation using either slip or stick boundary conditions in the Stokes drag factor, *μ*_e,pred_ = *q*/(*Cη*_eff_*a*), where *q* is the molecular charge, *a* is the translational hydrodynamic radius of a given dye (see Methods), *η*_eff_ is the effective viscosity of the liquid outside the molecules in the porous gel, and *C* = 4π for slip or *C* = 6π for stick boundary conditions. Since the average pore size of the gel^54^ is much larger than the size of all dye molecules, we assume *η*_eff_ ≈ 1 mPa s (i.e., the viscosity of water at room temperature) in *μ*_e,pred_, and we find that stick boundary conditions yield better overall agreement with *μ*_e,meas_.

### Visualizing complex formation between colliding dye pulses

**Fig. 4:**
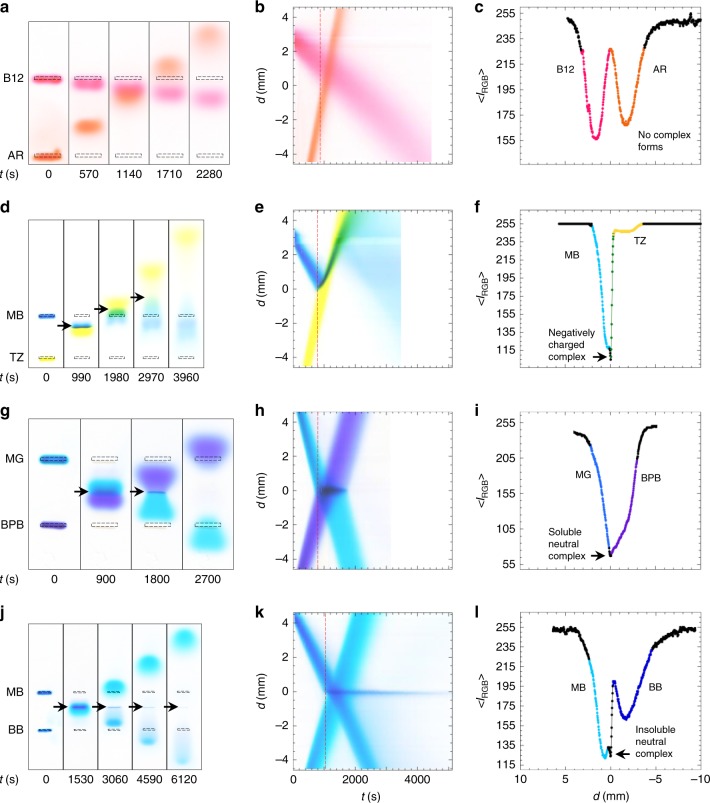
Optical visualization of complexing and decomplexing reactions between counter-propagating bands of organic dyes using BCGE. Conditions: 3.0% (w/w) agarose gel concentration, 5.0 mM sodium borate buffer at pH 9.0; electric field strength *E* = 3.1 V cm^−1^. Each well is loaded with 4 µL of a dye solution at 4.5 mM and measures 4 -mm wide. Shown for dyes B12 and AR: **a** background-subtracted overhead images at different times *t* after turning on *E*; **b** space–time plot of the center strip of pixels in the lane from part **a**, where the spatial position *d* along the lane is set to zero at the collision point; and **c** average RGB image intensity profile (corresponding to red dashed line in part **b**); lines guide the eye. The results for dyes MB and TZ are similarly shown in **d**, **e**, **f**; dyes MG and BPB in **g**, **h**, **i**; and dyes MB and BB in **j**, **k**, **l**. Arrows indicate complex formation. The *x*-direction points downward in spatial plots. Colors in parts **c**, **f**, **i**, and **l** are approximate guides only. Figure 2 defines dye abbreviations. Horizontal axes for **b**, **e**, **h** are the same as in **k**; horizontal axes for **c**, **f**, **i** are the same as in **l**. Source data are provided as a Source Data file

To explore interactions between dye molecules, as examples, we have performed BCGE on four dye pairs, B12/AR, MB/TZ, MG/BPB, and MB/BB (see Fig. 2) at a fixed 1:1 stoichiometric ratio of 4.5 mM initial dye concentrations (Fig. 4), chosen to be large enough to make attractive associations between dye molecules easy to visualize. Bands of neutral B12 and anionic AR simply pass through each other unperturbed (Fig. 4a; Supplementary Movie 3), indicating negligible attractive interactions. We construct a space–time plot by concatenating vertical strips of pixels from the center of a given lane for each recorded image (see Methods), and we find a constant velocity of propagation for both B12 and AR before, during, and after collision (Fig. 4b). At the onset of band collision, intensity analysis of this central vertical strip of pixels also reveals two rounded minima in the intensity without additional sharper features (Fig. 4c). By contrast, the other pairs show very different behavior. For instance, when TZ(−3*e*) collides with counter-propagating MB( + *e*), it actually reverses the propagation direction of nearly all MB ions, indicating strongly attractive charge interactions leading to predominantly anionic complex formation (Fig. 4d; Supplementary Movie 4). This is highlighted by the very different appearance of the associated space–time plot (Fig. 4e), showing a zig–zag pattern for MB, and the intensity analysis at onset of collision, which reveals a spike-like minimum (Fig. 4f), indicating complex formation. Subsequently, these net anionic complexes dissociate as they are continuously subjected to thermal-entropic Brownian fluctuations in the presence of the electric field. Ultimately, these stochastically excited complexes dissociate, and the dissociation products are separated by the electric field, yielding a smear in the space–time plot at longer times, which indicates that this reaction is reversible. The collision of BPB(−2*e*) and MG( + 2*e*), which involves a 1:1 charge ratio and nearly the same |*μ*_e,meas_|, also shows strong evidence of complexing by generating a dark stationary band (Fig. 4g, arrows). At later times, this band slowly disappears, as the combination of thermal-entropic fluctuations cause decomplexing, and the electric field pulls apart and separates these dye ions, yielding a symmetric two-color butterfly pattern. This dark band appears prominently as a persistent horizontal region in the space–time plot (Fig. 4h), corresponding to a spike-like minimum in the intensity after the onset of collision (Fig. 4i). As yet a different example, when a band of BB(−2*e*) collides with MB( + *e*), nearly all of the MB is consumed in a complexing reaction that generates a very long-lived stationary band, whereas a significant fraction of the more highly charged BB propagates through the collision without reacting (Fig. 4j–l). Thus, MB is the limiting reagent in this case. The very long lifetime of this stationary band may indicate not only that neutral complexes are formed but also that these complexes can aggregate locally into larger precipitates. Over time, this stationary band very slowly disappears, indicating that dissolution of the precipitates as well as dissociation of the complexes is likely occurring.

### Effect of reagent concentrations loaded at fixed volume

**Fig. 5:**
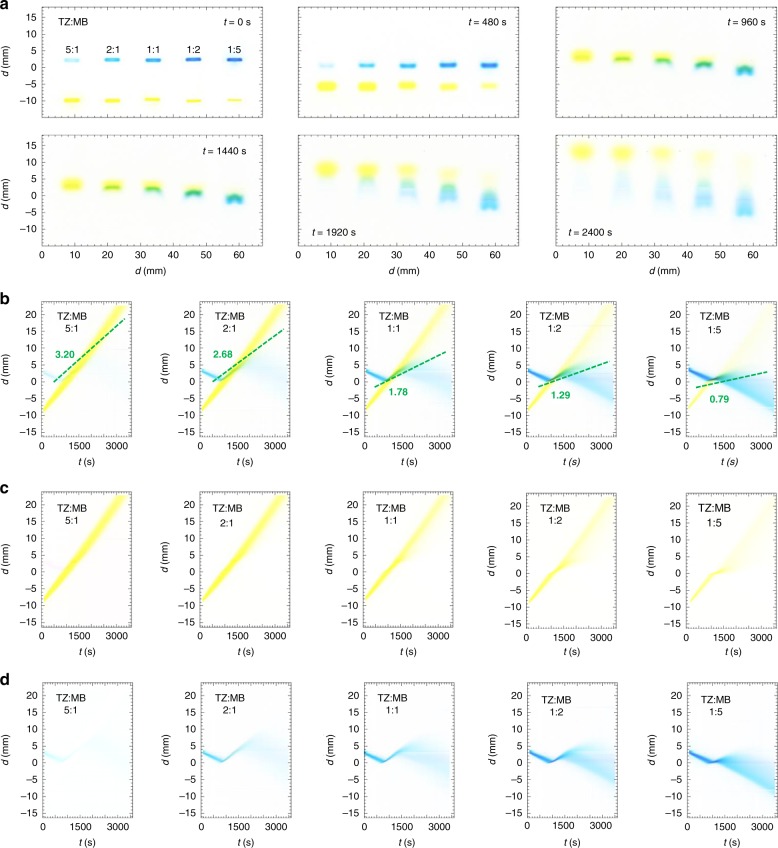
Concentration dependence of band collisions between complexing dyes that have different charge magnitudes using BCGE. Conditions: same as in Fig. 3. Separation between the two wells: *L* = 12 mm. Shown for dyes TZ(−3*e*, lower yellow bands) and MB( +*e*, upper blue bands): **a** background-subtracted overhead images at times *t* = 0, 480, 960, 1440, 1920, and 2400 s after turning on *E*. Upper left: lanes are marked according to concentration ratio TZ:MB at a fixed total concentration of 9.0 mM. **b** Space–time plots showing band trajectories of both TZ and MB. Offset green dashed lines: average trajectories of complexes containing MB immediately after collision; electrophoretic mobilities of these complexes *μ*_e,meas_ (green numbers) in units of 10^−8^ m^2^ V^−1^ s^−1^. Separation of color channels yields an optical absorption space–time plot of: **c** only TZ; and **d** only MB. The *x*-direction points downward in all plots

To more thoroughly investigate the temporary reversal in the propagation of the band of MB( + *e*, blue) when it encounters the faster moving and more highly charged counter-propagating band of TZ(−3*e*, yellow), we have performed BCGE for different molar ratios (TZ:MB) of these reagents ranging from 5:1 to 1:5 at a fixed total concentration of 9.0 mM (Fig. 5; Supplementary Movie 5). As the molar ratio increases toward higher [MB], the propagation of TZ is more significantly retarded (Fig. 5a), and space–time plots reveal nonlinear features in TZ, not just MB (Fig. 5b). By separating color channels (see Methods), we also make space–time plots of TZ only (Fig. 5c) and MB only (Fig. 5d). In contrast, for ratios 5:1 and 2:1, where TZ is dominant both in terms of concentration and charge per molecule, the trajectory of the band of TZ in its space–time plot is not significantly deflected (Fig. 5c). The duration over which MB propagates in the reverse direction after collision is longest at 5:1, and the slope of its trajectory while propagating as a complex with TZ is highest in the space–time plot at 5:1 (Fig. 5b). These slopes of complexed MB trajectories can be understood to represent the mobilities of the complexes formed, which decrease systematically as the molar ratio varies from 5:1 to 1:5 (Fig. 5b, dashed lines), indicating that the complexes, on average, are less negatively charged toward 1:5 (i.e. as [MB] significantly exceeds [TZ]). The post collision decomplexing behavior leads to significant dispersion and smearing of the bands as they begin to separate and ultimately cease interacting. A similar relative concentration dependence is observed for complexes of MG( + 2*e*) and AR(−2*e*), which for an equimolar collision forms a stationary complex which dissociates in equal proportions for MG and AR (Supplementary Movie 6). Based on these results, it is clear that the charges and mobilities of reagent species in combination with their relative concentrations and loaded volumes contribute to the complex pattern formation that is seen in BCGE.

### Influence of *E* on complexing and decomplexing

**Fig. 6:**
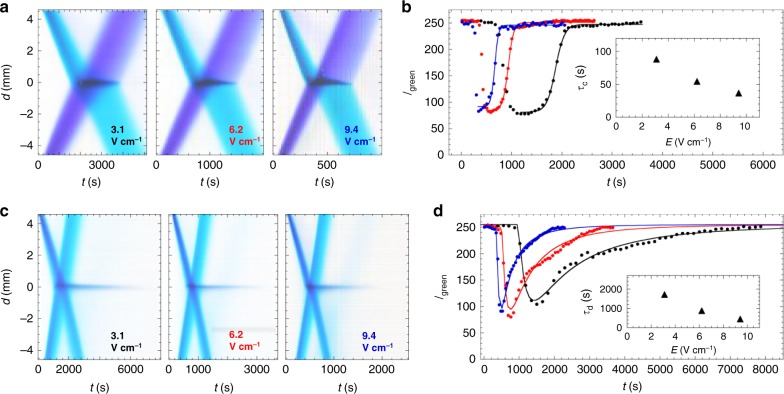
Dependence of complexing and decomplexing kinetics on the applied electric field strength using BCGE. Conditions: same as in Fig. 3, except *E*. **a** Background-subtracted space–time plots of band collisions between MG( + 2*e*) and BPB(−2*e*) at field strengths *E* (V cm^−1^) of 3.1 (black text, left); 6.2 (red text, middle); and 9.4 (blue text, right). The range of times shown for 6.2 and 9.4 V cm^−1^ have been reduced by factors of 2× and 3×, respectively, yielding similar X-patterns to that shown for 3.1 V cm^−1^. **b** Intensity profiles showing decomplexing kinetics, extracted from part **a** using the green color channel intensity *I*_green_ taken across *d* = 0 mm, where the stationary product band appears, as a function of time *t* for different *E* (points color coded as in part **a**); lines are fits of long-time data using Fermi-like functions (see Supplementary Table 2). Inset: time constant *τ*_c_(*E*) obtained from the fit decreases as a function of *E*. **c**, **d** Same as parts **a** and **b**, but instead for dyes MB(+*e*) and BB(−2*e*). In part **d**, *I*_green_(*t*) are fit using a modified log-normal function describing the optical absorption of the stationary band at *d* = 0 (lines, see Supplementary Table 3). The *x*-direction points downward in parts **a** and **c**. Inset: *τ*_d_ from the fits decreases for larger *E*. Source data are provided as a Source Data file

We have also investigated the lifetimes of the stationary complexes formed when MG/BPB and MB/BB are collisionally reacted at different *E* (see Supplementary Movies 7 and 8, respectively). As *E* is increased from 3.1 to 9.4 V cm^−1^, the total lifetimes of both complexes formed decrease (Fig. 6). For both MG/BPB and MB/BB collisions, rescaling the time axis in the space–time plots by the ratio of the field strengths leads to a universal appearance for each pair (Fig. 6a and c, respectively). The green intensity channel, *I*_green_, can be used to characterize the degree of optical absorption of attractive complexes of both pairs of dyes present in the stationary product bands, so we extract *I*_green_ at the position associated with the product bands, *d* = 0 mm, in the space–time plots (Fig. 6b and d, see Methods). The post collision long-time behavior of *I*_green_(*t*) for MG/BPB can be well described using a semi-empirical Fermi-like function that rises to a plateau (Fig. 6b; Supplementary Table 2), yielding a decreasing time constant *τ*_c_(*E*) associated with the spread of this Fermi-like function (Fig. 6b- inset). In contrast, the entire *I*_green_(*t*) for MB/BB is reasonably captured by a modified log-normal function that describes the optical absorption of the stationary band (Fig. 6d; Supplementary Table 3). While the shape of the log-normal is effectively independent of *E* (see *σ* in Supplementary Table 3), the time constant *τ*_d_ most closely associated with decomplexing decreases with *E*, but overall has a much higher magnitude (Fig. 6d, inset) than *τ*_c_; thus, MB/BB complexes are much more persistent than MG/BPB complexes.

### Band-collision gel electrophoresis in acidic buffer

**Fig. 7:**
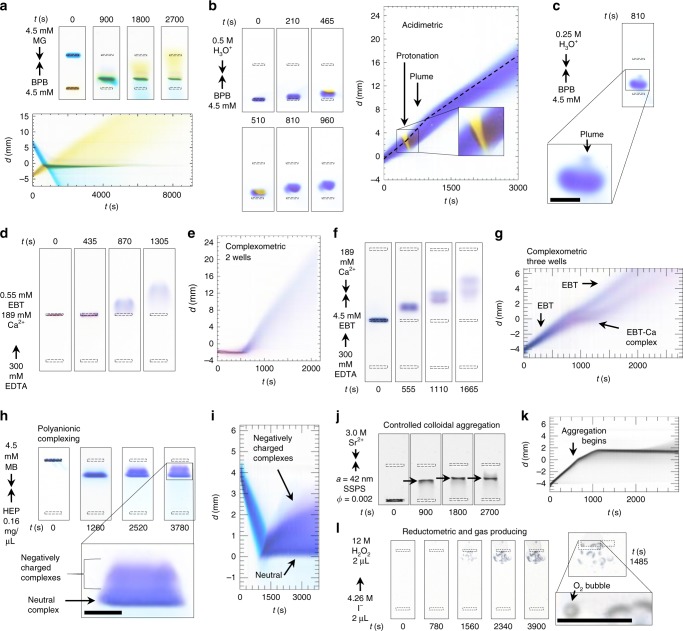
A wide variety of chemical reaction types are accessible using BCGE. **a** Protonated BPB(−*e*) collides with MG( +2*e*) in 5.0 mM CAA buffer at pH = 2.87 and *E* = 3.1 V cm^−1^. **b**, **c** Acidimetric band collisions between counter-propagating acid indicator dye BPB(−2*e*) and hydronium H_3_O^+^. **b** Overhead lane images and space–time plot revealing protonation of the leading edge of the band of BPB (yellow region) and subsequent ejection of a deprotonated BPB plume (purple region); **c** overhead lane image indicating less protonation of BPB, but more prominent plume ejection; **d**, **e** Two-well and **f**, **g** three-well complexometric ligand-exchange reactions between EBT, Ca^2+^, and EDTA (see the text). **d** Overhead lane images of a two-well complexometric reaction. Neutral EBT-Ca complex formed from EBT and Ca^2+^ remains in its well; Ca^2+^ is exchanged during EDTA collision, liberating EBT; **e** space–time plot of two-well ligand-exchange reaction. **f** Overhead lane images of three-well complexometric reaction where first Ca^2+^ collides with EBT forming the neutral complex before EDTA catches up and liberates the EBT; **g** space–time plot of three-well complexometric reaction. **h** Overhead lane images of HEP-MB collision resulting in both neutral complexes and partially negatively charged complexes, revealing spectral differences between these; **i** space–time plot of HEP/MB complexing reaction. **j** Overhead lane images of a controlled colloidal aggregation collision between Sr^2+^ cations and sulfate-stabilized polystyrene nanospheres (aggregates indicated by arrow); **k** space–time plot of the resulting colloidal aggregation reaction. **l** Redox reaction producing O_2_ gas bubbles after a band of propagating I^−^ anions collides with a band of stationary neutral H_2_O_2_ molecules. Scale: wells are indicated by dashed-line rectangles of 4.0 × 0.5 mm. Black scale bars in magnified insets: 2 mm. Unless otherwise stated, 4 μL of sample is added to every well. Conditions: same as in Fig. 3 unless otherwise noted. In all plots, the *x*-direction points downward

Although we typically use basic SBB at pH = 9.0, BCGE can also be performed in neutral and acidic buffers. As an example, we use a 5.0 mM chloroacetic acid (CAA) buffer at pH = 2.87, well below the first p*K*_a_ of BPB, so singly protonated BPB(−1*e*) in CAA buffer appears yellow, not blue, yet remains negatively charged. BCGE of BPB(−1*e*) with MG( + 2*e*) at this pH still results in complex formation (Fig. 7a; Supplementary Movie 9), yet the complex’s stoichiometric ratio in acidic CAA buffer differs from that in basic SBB.

### Acidimetric reaction of indicator dye with hydronium ions

Collision of an acidic pulse with a band of dye molecules, each of which has at least one site suitable for protonation, can still be performed even in a low-concentration basic buffer. We collide an invisible pulse of H_3_O^+^ with a 4.5 mM counter-propagating band of BPB, which serves as a pH-dependent color indicator dye, at two different high H_3_O^+^ concentrations, 0.5 and 0.25 M (Fig. 7b, c, respectively). These concentrated acidic pulses temporarily overcome the 5.0 mM SBB locally, but the H_3_O^+^ gradually becomes neutralized since the surrounding buffer has much greater volume, so these acid pulses become weaker over time. Upon collision, initially only the leading edge of the BPB band becomes singly protonated, revealing its yellow form (Fig. 7b, inset). Following this, a narrow faster-propagating plume of blue BPB is ejected, strikingly, in front of the rest of the band immediately after the protonated BPB returns to a deprotonated state (Fig. 7c, inset). A space–time plot of the collision between 0.5 M H_3_O^+^ and 4.5 mM BPB (see Supplementary Movie 10, middle lane) reveals several stages associated with different BPB propagation velocities (Fig. 7b). At the onset of collision, protonation, and the appearance of a yellow color, the BPB band slows. However, the rest of the band behind the yellow front continues to propagate, leading to a local build-up in concentration of protonated and deprotonated BPB. When the leading edge deprotonates because of buffer neutralization, the local charge density along this leading edge has become much higher, leading to an instability that propels the plume of BPB forward at a higher speed and with some spatial focusing. Eventually, local ionic concentrations re-equilibrate to near their original values, and the original mobility of BPB returns.

### Complexometric ligand-exchange reactions

A common indicator used for complexometric titrations is Eriochrome Black T (EBT), which dissociates in water yielding anions having −1*e* charge and a blue appearance^55^. However, when complexed with Ca^2+^, EBT has a pink appearance^56^. We perform a BCGE experiment demonstrating displacement in which the strong divalent-cation chelator ethylenediamine–tetraacetic acid^57^ (EDTA^4−^) strips Ca^2+^ from more weakly bound EBT–Ca complexes^58^. EDTA–Ca complexes will have net negative charge because of the excess negative charge in the EDTA^4−^ compared with Ca^2+^. The chelation capacity of EDTA is greatly reduced when one Ca^2+^ is bound, so the likelihood of neutral EDTA–2Ca complexes being formed and remaining stable is extremely low. While we do not visualize the invisible EDTA–Ca complex, it would migrate as a negatively charged complex. We load 0.55 mM EBT with 189 mM Ca^2+^ into one well (Fig. 7d, top well), and we load invisible 300 mM EDTA into a second well in the same lane (Fig. 7d, lower well). Initially, the pinkish EBT–Ca complexes do not propagate and remain in the top well, yet EDTA propagates from the bottom well toward the top well, and it eventually collides with the EBT–Ca complexes, liberating blue EBT^−^. The corresponding space–time plot (Fig. 7e) reveals the change in a stationary pink band of EBT–Ca complexes after EDTA collides with it, strips and chelates Ca^2+^ to form an invisible EDTA–Ca complex, and thereby liberates EBT^−^ which becomes a propagating blue band.

### Programmed chemical reactions of colliding bands

To demonstrate the flexibility of BCGE for creating more complex sequences of reactions, a third well in the same lane creates a sequential reaction (Fig. 7f). A band of invisible Ca^2+^ collides with a band of blue EBT^−^, yielding some pink EBT–Ca complexes, and then invisible EDTA collides with this EBT–Ca complex, thereby stripping off and chelating the Ca^2+^, so EBT changes back to a blue color and resumes anionic propagation. Corresponding to this more complex reaction sequence using three wells, the space–time plot also has significantly greater complexity (Fig. 7g).

### Additional demonstrations of the broad utility of BCGE

We have changed the charge state of poly-ionic biopolymers, irreversibly aggregated nanospheres to halt their propagation in the gel, created gas bubbles through a redox reaction, and collided invisible bands of anionic and cationic surfactants with visible bands of oppositely charged dyes. For instance, we collide a slower-moving band of invisible poly-anionic heparin^59^ (HEP) with a faster-moving band of blue MB(+*e*) to form purple HEP–(MB)_*x*_ complexes^60^ (Fig. 7h). Some HEP molecules are fully neutralized by MB and stop propagating, yielding a purple stationary band, whereas other HEP molecules are only partially neutralized to different degrees and continue propagating to form a purple-blue smear (Fig. 7i; Supplementary Movie 11). We have also used sulfate-stabilized polystyrene (SSPS) nanospheres as reagents in a passivated gel form of BCGE; the nonionic passivation agent PEG-1000 has been added at 3.25 mM to allow SSPS nanospheres to propagate in a large-pore agarose gel^45,61^ (see Methods). Sulfate groups on a sphere becomes irreversibly bound to sulfate groups on other spheres, mediated by Sr^2+^ cations^62^, leaving behind a narrow stationary band of nanosphere aggregates larger than the gel’s pore size (Fig. 7j, arrows and Fig. 7k, dark horizontal line). In addition, we show that a redox reaction^63^ of a propagating band of iodide I^−^ (2 µL of 4.26 M loaded) with a stationary band of neutral hydrogen peroxide H_2_O_2_ (2 µL of 12 M loaded) results in the formation of excess insoluble O_2_ gas liberated as visible bubbles in the H_2_O_2_ well-region (Fig. 7l), according to a slower reaction that produces the intermediate species IO^−^(*aq*)

1 $${\mathrm{H}}_{\mathrm{2}}{\mathrm{O}}_{\mathrm{2}}\left( {aq} \right) + {\mathrm{I}}^-\left( {aq} \right) \to {\mathrm{H}}_{\mathrm{2}}{\mathrm{O}}\left( l \right) + {\mathrm{IO}}^-\left( {aq} \right)$$

followed by a rapid reaction that generates gaseous oxygen

2 $${\mathrm{H}}_{\mathrm{2}}{\mathrm{O}}_{\mathrm{2}}\left( {aq} \right) + {\mathrm{IO}}^-\left( {aq} \right) \to {\mathrm{H}}_{\mathrm{2}}{\mathrm{O}}\left( l \right) + {\mathrm{O}}_{\mathrm{2}}\left( g \right) + {\mathrm{I}}^{\mathrm{-}}\left( {aq} \right).$$

Some bubbles become trapped within the gel. Although one reagent is charge-neutral, the other is not, so the relative difference in propagation velocities still enables these bands to be collided and reacted. If the reaction product is visible, then simple optical imaging can still be used to detect and quantify the extent of the reaction, even if both bands of reagents are invisible. If two invisible bands propagate and collide to produce a visible product, then optical BCGE can be used to directly measure *μ*_e_ of the reagent species through the location of and elapsed time until collision. As additional examples, we have created stationary complexes by colliding invisible anionic surfactant dodecyl sulfate DS^−^ with cationic dyes MAL, MB, and MG (Supplementary Movie 12), and by colliding invisible cationic surfactant dodecyl trimethylammonium DTA^+^ with anionic dyes TZ, AR, BB, BPB, and BCG (Supplementary Movie 13).

## Discussion

Optical BCGE provides detailed movies of spatiotemporal pattern formation associated with collisional reactions of solvated and propagating reagent species in spatially localized bands within gels. Highly mobile reactant, complex, or product species can be contained in inert environments, thereby extending observation periods where fast kinetics could otherwise not be studied. Similar to gas-phase investigations utilizing matrix-isolation methods^64,65^, BCGE is well-suited for studying both reversible and irreversible reactions involving only small quantities of reagents, and product species can typically be separated and isolated from unreacted reagents in situ. Moreover, complex sequences of reactions of bands can be effectively programmed by designing the locations of multiple wells in the same lane, similar to the programmability of flow-driven microfluidic channel systems. Thus, BCGE offers significant advantages over a single-well non-collisional EMSA when performing GE on interacting species.

Predicting the often striking, yet complex, evolving spatiotemporal patterns created by BCGE represents an interesting challenge for theoretical modeling and simulation. Such predictive modeling would need to incorporate and appropriately couple many different physical and chemical effects, over and above the electrophoretic propagation of species in an electrolyte buffer at a certain pH within a porous gel. These effects include forward and reverse reaction rates, effective collisional cross-sections of reagent species, Brownian diffusion of propagating molecular and colloidal species, reaction stoichiometry, diversity of product species, potential growth of products produced as aggregates or precipitates relative to the gel’s pore size, electric field strength, pH-dependent conformations and ligand-dependent conformations, and spectroscopic properties of molecules. Theoretical advances could lead to predictions of space–time plots complete with full spectroscopic detail, which could be quantitatively compared with BCGE measurements. Nevertheless, the complex inverse problem of solving for fundamental intermolecular interaction parameters as well as types and structures of product species from the evolving patterns of BCGE is likely at least in some cases to be ill-posed mathematically, and a unique solution to this inverse problem is not guaranteed. Despite this, it is also likely, at least initially for certain simpler reagent and product species, that it will be possible to model the spatiotemporal patterns in BCGE and thereby extract useful molecular-scale interaction parameters from measurements. Greater degrees of complexity could then be introduced into the reagent species, reaction types, and modeling, further extending the range of quantitative interpretation of BCGE.

We anticipate that many interesting experimental applications and extensions of BCGE lie ahead (see Supplementary Discussion). For instance, BCGE is not inherently limited to a visible color array detector; it could be extended to wavelengths beyond the visible spectrum. Using fluorophore-labels, quantum dots, photonic nanoparticles, or other absorbing stains in combination with, for instance, ultraviolet illumination and visible detection wavelengths could reveal certain propagating bands that would otherwise be invisible. Using a variety of different optical configurations, modalities, and wavelengths, rather than just visible absorption in a transmission geometry, we anticipate that BCGE can be extended to visualize interactions between biomacromolecules, including proteins and poly-nucleic acids. Spatially resolved spectroscopies could be used to measure concentrations of reactants, products, and long-lived intermediates more precisely. Also, BCGE can be readily generalized to pH-neutral and acidic buffers. Moreover, we anticipate that 2D and 3D versions of BCGE will provide access to even higher levels of complex programmable reactive combinatorial chemistry coupled to electrophoretic separations.

## Methods

### Gel and dye preparation

All gels are prepared using Sigma-Aldrich Type I-A, low EEO agarose at 3.0% w/w in distilled water (conductivity measured to be <0.5 μS cm^−1^)^45^. Using this agarose concentration corresponds to characteristic pore sizes of the gel of ≈50 nm, since nanospheres having diameters smaller than ≈50 nm will propagate through the gel^61^. This higher gel concentration also reduces diffusion of dye molecules, lowering dispersion (i.e. width of bands). For experiments involving polystyrene nanospheres, we use a considerably lower gel concentration of 0.195% w/w, yielding a characteristic pore size of ≈300 nm. When making dye solutions, we follow common GE protocols by adding D_2_O, which has a higher mass density than H_2_O and therefore causes the solution containing the dye molecules to sink to the bottom of the wells prior to turning on the electric field. This provides better uniformity in the vertical location of the loaded dyes in the wells prior to migration and collision. Dyes (see Supplementary Table 1 for manufacturer and purity) are dissolved in distilled water before diluting 1:1 with D_2_O (Cambridge Isotope Laboratories, Inc., 99% purity) to a final dye concentration of 4.5 mM unless otherwise indicated. High reagent concentrations greater than 1 M (e.g. in Fig. 7) are sufficiently higher in density than the surrounding buffer, and are not mixed with D_2_O before loading.

### Gel electrophoresis

We use a transparent acrylic horizontal GE apparatus (see Fig. 1a) with Pt electrodes (American Scientific, LLC, item 8101–00); chamber dimensions are 152 × 76 × 44 mm, and each gel slab is 70 -mm wide, 100 -mm long (i.e., along the electric field), and 3.5 -mm thick. We make custom gel combs by laser cutting thin poly-tetrafluoroethylene (PTFE) sheets to create 4.0 × 0.5 mm wells that are 2.5- mm deep. We control distances between wells in the same lane using two or more combs when casting. The chamber is filled with buffer solution (typically 5.0 mM sodium borate buffer at pH = 9.0) to a height of 2.5 cm above the gel surface. While we report electric field strengths to enable comparison with other GE experiments, we operate our power supply in constant current mode throughout all experiments to keep band velocities constant over longer durations^45^. All infusions of samples into wells are 4 μL in volume unless otherwise stated. Wells are loaded from left to the right, top to the bottom. To reduce initial diffusion of the dyes after loading into the wells in the porous gel, the power supply is activated as soon as all wells have been filled, so that no reagent has been in a well more than 30 s after loading before the electric field is applied.

### Minimizing interactions with the porous gel matrix

The hydrodynamic radius of a typical organic dye molecule used in this investigation is significantly smaller than the characteristic pore size of the agarose gels used, such that interactions between such dye species and gel are largely negligible, particularly when compared with those that can occur between large biomacromolecules confined to the typically much smaller pores of polyacrylamide gels during EMSAs. Reagent dye molecules and small-product complexes of these dyes are likely to experience only minimal gel-matrix effects on their transport properties in the large-pore agarose gels that we use. Reaction kinetics and propagation velocities of bands can depend on pore size if the sizes of reagents and/or products are not much smaller than the gel’s characteristic pore size. We present aggregation reactions that demonstrate this limit, both with molecular and colloidal reagents, since the product aggregates exceed the pore size and subsequently do not propagate.

### Image acquisition

All images are taken using a Nikon D5000 DSLR camera body equipped with a Nikon 70 mm−300 mm zoom lens set at ≈195 mm and rigidly mounted ≈1 m above the GE tank. Gels are illuminated from underneath the tank by a light box (CubeTech HL225 natural/white LED at 10,000 lux), yielding a transmission optical format. Exposure settings are ISO 200, f/10, and 1/60 s. Pixel saturation is avoided by reducing the exposure time, if needed. Single frames (4288 × 2848 pixels, RGB 24-bit color) are taken every 15 s unless otherwise indicated, yielding time-lapse movies. Color balance is calibrated using a 24-color card and standard procedures (CameraTrax). Reference background images are taken immediately prior to loading any wells.

### Background subtraction of images

To increase signal-to-noise, we subtract the corresponding reference background image (prior to loading) from each measured image in a sequence using the following procedure (ImageJ). After inverting the image from the sequence, an inverted background image is subtracted using image calculation, and the resulting background-subtracted image sequence is inverted again. This double inversion is appropriate for background-subtracting transmission images of absorbing dyes.

### Extracting intensity profiles and making space–time plots

A single-pixel strip along the field direction (i.e., vertical) in the center of a lane is extracted from an image using MATLAB, and intensity values in each RGB channel are quantitatively determined. For a given lane, successive vertical pixel strips are extracted from an image sequence and concatenated horizontally, yielding a space–time plot that summarizes quantitative spatiotemporal evolution of band collisions and reactions.

### Time and location of a band collision

We consider BCGE involving two wells in the same lane (see Fig. 1b); the first is centered at position *x* = 0, and the second is centered at *x* = *L*. Reagent species 1 and 2 have electrophoretic mobilities *μ*_e,1_ and *μ*_e,2_, respectively. These reagents are initially loaded into these wells in the absence of an electric field. Having prior knowledge of the values (or at least the predicted signs at a given pH) of *μ*_e,1_ and *μ*_e,2_ is typically desirable, since this is useful in choosing which reagent species to load into a specific well, given the direction of the electric field *E*, and also in selecting an appropriate value of *L* in order to generate a collision of propagating bands in the gel. However, if *μ*_e,1_ and *μ*_e,2_ are not known in advance, both combinations of loading can be performed in two different lanes (i.e., species 1 in a well at *x* = 0 and species 2 in a well at *x* = *L* in a first lane, and vice versa in a different lane); this will typically yield a collision of bands in only one of the two lanes. In what follows below, we assume that reagent species 1 is loaded into the well at *x* = 0, and reagent species 2 is loaded into the well at *x* = *L*. The electric field *E* is turned on at time *t* = 0, and the centers of the bands of reagent species 1 and 2 propagate to positions *x*_1_(*t*) = *μ*_e,1_*Et* and *x*_2_(*t*) = *L* + *μ*_e,2_*Et*, respectively, at a time *t*. These equations assume that *E* is spatially homogeneous and remains constant over time. Solving these equations for the collision of the centers of the bands, which occurs when *x*_1_(t*) = *x*_2_(t*) = x*, we determine the collision time t* = *L*/[(*μ*_e,1_ – *μ*_e,2_)*E*] and collision location x* = *L*[*μ*_e,1_/(*μ*_e,1_– *μ*_e,2_)].

Several different scenarios for generating collisions of bands are possible using BCGE (see Fig. 1c–e). In the most common scenario, which we call counter-propagating BCGE, the collision location always occurs between the two wells; the electrophoretic mobilities of the two reagent species must have opposite signs, and must be loaded into the appropriate wells, such that *μ*_e,1_>0 and *μ*_e,2_<0. This ensures that the difference *μ*_e,1_−*μ*_e,2_, which appears in the denominators of the expressions for t* and x*, is always positive. Two other scenarios, which we collectively call uni-propagating BCGE, involve reacting a charged reagent species with an uncharged one. If the charged reagent species has a positive charge, then we consider it to be reagent species 1 such that *μ*_e,1_ > 0, and it is loaded into the well at *x* = 0; the uncharged reagent species 2, which therefore has *μ*_e,2_ = 0, is consequently loaded into the well at *x* = *L*. Because the uncharged reagent species does not propagate when the field is turned on (or possibly displaces only slightly as a consequence of potential electro-osmotic effects), the collision point of the bands will be x* ≈ *L*. By contrast, if the reagent species has a negative charge, then we consider it to be reagent species 2 such that *μ*_e,2_ < 0 and it is loaded into the well at *x* = *L*; the uncharged reagent species 1, which therefore has *μ*_e,1_ = 0, is consequently loaded into the well at *x* = 0. Because the uncharged reagent species does not propagate when the field is turned on (or possibly displaces only slightly as a consequence of potential electro-osmotic effects), the collision point of the bands in this case will be x* ≈ 0. Alternatively, in yet a different scenario, which we call co-propagating BCGE, *μ*_e,1_ has the same sign as *μ*_e,2_, yet their magnitudes are different such that *μ*_e,1_ ≠ *μ*_e,2_. In this case, the difference *μ*_e,1_−*μ*_e,2_, which appears in the denominators of the expressions for t* and x*, is nonzero and a collision can still occur, yet the value for x* corresponds with a location that is outside of the region between the two wells. For co-propagating BCGE, care must be taken in selecting a sufficiently small value of *L* so that the collision location x* occurs inside the physical boundaries of the gel.

The above equation for the collision time t* clearly reveals that it becomes impossible to generate a collision between bands when *μ*_e,1_ = *μ*_e,2_, since t* effectively diverges and becomes infinite in the limit as *μ*_e,1_ approaches *μ*_e,2_. Even if *μ*_e,1_ ≈ *μ*_e,2_, yet the two electrophoretic mobilities are not strictly equal, it can become practically impossible to generate a collision of bands within the physical boundaries of the gel, even if *L* is chosen to be very small. Thus, for *μ*_e,1_ ≈ *μ*_e,2_, the only viable approach that can enable the reagent species to interact is to load them in the same well initially, generating the limiting case of the electrophoretic mobility shift assay (EMSA).

As an example of a co-propagating collision of bands created using BCGE (see Fig. 1e for schematic), we have loaded the first well at *x* = 0.0 mm with AR dye (*μ*_e,1_ = −2.75 × 10^−8^ m^2^ V^−1^ s^−1^) and the second well at *x* = *L* = 5.0 mm with BB dye (*μ*_e,2_ = −1.68 × 10^−8^ m^2^ V^−1^ s^−1^). At pH = 9.0, both dyes are negatively charged, so their electrophoretic mobilities have the same sign, but significantly different magnitudes. As predicted, the collision of the two different bands of dyes occurs in the gel outside of the region between the two wells at x* ≈ −12.8 mm. In this experiment, no interaction between the colliding bands was observed, and the space–time plot reveals no deviations in propagation of the dyes or additional streaks indicating complex formation as a consequence of the collision. We reason that this is because electrostatic interactions between these like-sign small-molecule reagent dye species are dominantly screened-charge repulsions; so, no complex forms, even transiently. However, this single result does not imply by itself that other molecular types, such as those that have long hydrophobic chains in addition to charge, would not visibly react or associate in a co-propagating BCGE scenario. Likewise, some types of redox reactions involve two negatively charged species that have significantly different electrophoretic mobilities, and these reactions could potentially be probed as a function of reagent concentrations using co-propagating BCGE.

### Estimating electrophoretic mobilities of invisible species

For invisible reagent species, if the average collision location x* and average time-to-collision t* associated with measurable evidence of product formation can be detected, then it is possible to determine the electrophoretic mobilities of both reagent species. Dividing the equation for x* by the equation for t* and solving, the electrophoretic mobility of the first reagent species is given by: *μ*_e,1_ = (x*/t*)/*E*. Using this result, the electrophoretic mobility of the second reagent is given by: *μ*_e,2_ = *μ*_e,1_−(*L*/t*)/*E*. These equations can also be used to determine the electrophoretic mobilities of visible reagents from the measured collision time and location; although these reagent mobilities could also be determined by tracking the velocities of the propagating visible bands before collision, too. Because of the potential for Brownian diffusion of reagent species in addition to electrophoretic propagation, the first evidence of product formation may not correspond precisely to t*, since the leading edge of the band is diffuse. So, when determining t* from product formation, it is typically best associated with the peak production of product species, not the first observable evidence of product species.

Using BCGE, if a visible or otherwise detectable reaction product is formed between colliding bands of two different reactant species, whether these reactant species are invisible or visible, then electrophoretic mobilities of both reactant species can be deduced from the location of the detected reaction product relative to the wells where the reactant species were loaded, the elapsed time associated with the maximum detected product formation after activation of the electric field, and the strength and direction of the electric field. As an example, we determine electrophoretic mobilities of invisible reactant species by performing BCGE between bands of visible dyes and invisible ionic surfactants. Bands of cationic dyes are collided with bands of DS^−^ (Supplementary Movie 12), and bands of anionic dyes are collided with bands of DTA^+^ (Supplementary Movie 13). Stationary, neutral complexes form when DS^−^ collides with MAL, MB, and MG, and also when DTA^+^ collides with AR, BB, BPB, and BCG. Given the locations and times of formation of the stationary bands of the neutral complexes, as well as the applied field strength, we estimate the velocities of the invisible reactant species and then determine that the values of *μ*_e_ for DS^−^ and DTA^+^ at these conditions are −1.1 × 10^−8^ m^2^ V^−1^ s^−1^ and +1.4 × 10^−8^ m^2^ V^−1^ s^−1^, respectively. In a different BCGE trial, we have also collided a band of invisible Sr^2+^ with a counter-propagating band of invisible DS^−^ to form the white insoluble precipitate Sr(DS)_2_, which is readily detected optically against a black background using side illumination of the gel rather than transmission illumination. Because the velocities of both invisible species can be determined by the distances of propagation from the loaded wells to the collision point, and the time associated with the collision and detected product formation, one can deduce the electrophoretic mobilities of both the invisible Sr^2+^ and the invisible DS^−^ reactant species.

### Modeling the effective hydrodynamic radii of dyes

We build molecular models of dyes (HyperChem Professional 8.0.7). We include charges on charge groups according to the acid/base chemistry of that particular dye at the given pH = 9.0 (see Supplementary Methods and Supplementary Fig. 1). The model is geometry-optimized in vacuo until convergence with a root mean-square gradient of <0.1 kcal Å^−1^ mol^−1^ using the Polak–Ribiere conjugate gradient algorithm^66^. Optimized geometries are placed in a 20 Å cubic periodic bounding box populated with ≈195–245 water molecules depending on the size of the dye. The minimum distance between solvent and solute is set at 2.3 Å. Molecular mechanics are calculated using the AMBER force field with bond, angle, torsion, non-bonded, electrostatic, and hydrogen-bonded components. Options include a constant dielectric of scale factor 1 (i.e., water), switched inner and outer cutoff radii of 6 and 10 Å, respectively, and 1–4 scale factors (nonbonded interactions separated by exactly three covalent bond distances) of 0.5 for electrostatic and van der Waals interactions to reduce the exaggerated short-range repulsion of the Lennard–Jones 6–12 potential. After convergence, water molecules are removed, and solvated dye structures (see Supplementary Fig. 1) are imported into WinHydroPRO v1.00 PUB (temperature *T* = 20.0 °C, solvent viscosity = 1.00 mPa s, partial specific volumes of ≈1.0 cm^3^ g^−1^). Hydrogen atoms are ignored. Atomic radii of non-H atoms in a bead model are assigned values of 2.84 Å (see prior publication^67^), resulting in an average van der Waals radius of ≈2.9 Å and a 1.1 Å hydration layer. These modeling results yield a hydrodynamic translational radius for each dye molecule, which is used in the Stokes drag factor to predict its mobility.

## Supplementary information

Supplementary Information

Peer Review

Description of Additional Supplementary Files

Supplementary Movie 1

Supplementary Movie 2

Supplementary Movie 3

Supplementary Movie 4

Supplementary Movie 5

Supplementary Movie 6

Supplementary Movie 7

Supplementary Movie 8

Supplementary Movie 9

Supplementary Movie 10

Supplementary Movie 11

Supplementary Movie 12

Supplementary Movie 13

Source Data

## Data Availability

The source data related to Figs. 3b, 4c, 4f, 4i, 4l, 6b, and 6d are available as a Source Data file with this paper (see Supplementary Information section online).
