# Supplementary material for: Band-collision gel electrophoresis
Source: Nat Commun. 2019 Aug 12;10:3631. doi: 10.1038/s41467-019-11438-9 (PMC6690962; doi:10.1038/s41467-019-11438-9)
Supplement: Supplementary file 1 — Supplementary Information [file 41467_2019_11438_MOESM1_ESM.pdf]

Supplementary Information

Band-collision gel electrophoresis

Dimitri A. Bikos[1] and Thomas G. Mason[1,2,*]

[1]*Department of Chemistry and Biochemistry,*

University of California- Los Angeles, Los Angeles, CA 90095 USA.

and

[2]*Department of Physics and Astronomy*

University of California- Los Angeles, Los Angeles, CA 90095 USA.

*Corresponding author: Thomas G. Mason; e-mail address: mason@chem.ucla.edu

Table of Contents

Supplementary Methods

Supplementary Discussion

Supplementary Figures

Supplementary Tables

Supplementary References

Supplementary Methods

Determining Total Charge on Molecular Ions

Molecular ions that are known to absorb visible light can be categorized according to the presence of structural motifs associated with their optical properties[1]. All anionic molecules used in this investigation contain at least one alkyl sulfonate group. Sulfonates are very strong acids with pK_a values less than zero[2] and for practical purposes can be assumed to remain deprotonated throughout the pH range in most experiments, including our study. The acid dissociation constants are available in the literature for the following dyes: tartrazine (TZ)[3,4], allura red AC (AR)[3], brilliant blue FCF (BB)[5,6], bromophenol blue (BPB)[7], bromocresol green (BCG)[8], cyanocobalamin (B12)[9], rhodamine B (RB)[10], malachite green (MAL)[11,12], methylene blue (MB)[13], methyl green (MG)[14], and neutral (RB, vitamin B12) dyes[15]. The slow cationic propagation of RB and B12 that we observe can be attributed to electroosmotic flow[16]. The molecular ions BB, BPB, BCG, MAL, and MG belong to the triarylmethane class. Two highly acidic tosyl groups extend the structure of BB, providing flexibility in distinguishing this molecular ion from others used in our study. BB has two reported pK_a values[6]: 5.83 and 6.58. The BB molecule is zwitterionic with a predicted overall charge of -2*e* in a pH = 9.0 solution. Structurally, BPB and BCG differ only by the presence of a methyl group in two of three aryl rings. BPB has a spectroscopically determined pK_a of 3.95 [7] associated with a widely-known color change from yellow to blue between pH 3.0 and 4.6, respectively. The phenolic oxygen remains deprotonated below this value[17] until a structural rearrangement occurs, which neutralizes the overall charge of the molecular ion[18]. A pK_a of 4.85 has been measured for BCG[8]. It is predicted that BCG exists in its monoanionic form below pH = 4.85, above which the molecular ion is dianionic[19]. MAL changes color from yellow to green, then green to colorless

between pH values of 0.0 to 2.0 and 11.6 to 14.0, respectively. Identification of a pK_a at 6.90 appears to correspond to a kinetically limited hydrolysis reaction involving a structural transition with an associated pK_{hyd} of 6.9 [12]. We observe no change in electrophoretic mobility for MAL below pH = 6.90 in our experiments. The pK_a of the amino group in MAL is 2.2 [11]. We predict that MAL has a +1*e* charge at pH = 9.0. MG undergoes a color change from yellow to greenish-blue at a pH of 0.1 to 2.3 and has a pK_a within the range of 0.2 - 1.8 [14]. Two quaternary nitrogens remain positively charged across a wide spectrum of pH imparting a charge of +2*e* at pH = 9.0. Many triarylmethane molecular ions are known to slowly form colorless carbinols at higher pH values[12]. MAL and MG have been reported to undergo this hydrolysis reaction at pH 9.0 [11]; however, time scales for this hydrolysis reaction are much longer than our typical observation times after loading and running gels, so we find that this potential effect is negligible in our study. We dissolve molecular ions in pure water (with some D_2O as described in the Methods) and perform experiments in under 1 hour to prevent accumulation of hydrolysis products.

Two azo-class molecules represented in this study are TZ and AR, characterized by the presence of functional group R-N=N-R'. TZ undergoes an azo-hydrazone tautomerization reaction characteristic of pyrazolones. The sulfonate and carboxylate moieties of TZ suggest the molecular ion can possess a charge of -3*e* across a wide range of pH values. It is only for pH > 10, that TZ exists predominantly in a tetra-anionic form[20]. Spectroscopic methods have measured the pK_a of TZ to be 9.4 [3], a value corroborated by fixed titration methods[4]. Despite the proximity of the pK_a of TZ to the pH of SBB, we observe best agreement to theory at a charge of +3*e*. TZ does not readily form aggregates or dimers in aqueous solution[21]. AR contains two negatively charged sulfonate groups with an azo group pK_a of 11.4 in aqueous solution[3]. In the range of pH investigated, AR is predicted to possess a charge of +2*e*.

MB is a heterocyclic dye of the thiazine class. The molecular ion of MB is a planar phenothiazine with a pK_a identified as 3.8 [13]. The predicted charge of MB at pH = 9.0 is +1*e*.

The cyanocobalamin vitamer B12 has a cherry color that is produced by the cobalt-corrin complex at its center. Vitamin B12 has three reported pK_a values corresponding to 1.0, 2.9, and 4.7 [9]. At a pH of 9.0, B12 should be fully neutral. Its presumed neutrality has made it a popular probe for measuring electroosmotic flow.

RB is a fluorone zwitterion with a pK_a of 3.1 [10]. Its carboxylate group is neutralized and deprotonated under pH of 3.1 giving the molecular ion a charge of +1*e*. Individual charges on the amine and carboxylate cancel at pH = 9.0 giving RB a net charge close to zero.

In our experiments, BB does not propagate as rapidly as its size and charge would predict, at least on first cursory inspection. However, a reasonable explanation may be found in the flexibility of the solvated BB zwitterion. Solvated geometry optimization predicts that attractions between charges within the BB molecule will result in a folded structure. Folding may increase charge screening. As most molecular dyes have similar sizes, it is likely that folding results in charge screening, which in turn lowers the propagation rate for BB. The indicators BPB and BCG also propagate more slowly than predicted. It may be possible that partial protonation leads to a slightly lower non-integer time-average charge for these molecular ions.

Using the predicted charges on charge groups at pH = 9.0, we have calculated structures of dye molecules, as shown in Supplementary Fig. 1 (see main Methods section for modeling software used and model parameters). Positive and negative signs indicate regions of appreciable local charge density. For clarity, water molecules are not shown. Intramolecular attraction between an anionic sulfate and a tertiary amine cation results in a folded structure in BB.

Supplementary Discussion

Alternative Illumination-Detection Modalities and Configurations for BCGE

While we have demonstrated the broad approach of band-collision gel electrophoresis (BCGE) through two different illumination-detection configurations involving optical absorption and scattering of visible light, BCGE is not inherently limited only to those two configurations and imaging modalities. In the transmission configuration/modality, we have illuminated bands of optically absorbing species (*e.g.* dyes) or refracting species (*e.g.* bubbles) with visible white light using a light box below the transparent gel electrophoresis (GE) chamber and a camera with lens above this chamber, thereby enabling us to image these species through reductions of transmitted light intensity at particular wavelengths. In the scattering configuration/modality, we have illuminated bands of optically absorbing, scattering, or refracting species with white light from the side (*i.e.* propagating predominantly in the plane of the gel at 90° with respect to the camera's optical axis) using one or two light boxes. In the case of the scattering configuration/modality, it is typically beneficial to place a black absorbing plastic film, paper, or cloth underneath the GE apparatus in order to enhance contrast of the light scattered by the scattering species towards the camera.

Beyond these two demonstrated illumination-detection configurations/modalities using white visible light, other configurations/modalities some of which may involve wavelengths beyond the visible spectrum, can also be useful for BCGE. For example, backscattering illumination of white light can be used, such that a light box or a fiber light is placed nearly in-line with the camera and pointed towards the gel (*i.e.* nearly along the optical axis of the camera's lens), so that the downward propagating light illuminates the GE apparatus and thus reagent/product species in the gel, and the camera collects upward propagating light that leaves

the gel traveling in substantially the opposite direction. Beyond transmission and side illumination, this backscattering configuration/modality can be used to image absorbing, scattering, and refracting species.

Beyond visible white light illumination, BCGE can be readily extended to a fluorescence configuration/modality for imaging fluorescent molecular and colloidal species and also fluorescently labeled molecular and colloidal species that would otherwise be invisible. For instance, for fluorescent molecules that emit light at a visible wavelength when illuminated with ultraviolet (UV) light, a transmission geometry involving a UV light box and optical filter, which removes any residual visible light emanating from the UV illumination, can be used. The material for the chamber of the GE apparatus is typically chosen to appreciably transmit UV light at the absorption wavelength of the molecular dye, so that illuminating through the bottom of the GE apparatus does not cause a large reduction in the intensity of UV light that reaches the fluorescent molecules. For instance, although UV-filtering acrylic is the predominant form of manufactured acrylic, UV-transmitting acrylic is available and can readily be made into GE chambers suitable for BCGE involving UV illumination through the chamber. Typically, in order to avoid or reduce bleaching of the fluorescent molecules, it is also desirable for the UV illumination to be on (*i.e.* active) only periodically, rather than continuously, and coordinated with the periods of time during which the shutter of the camera is open. In some cases, it can be desirable to use broad-band UV light, which can excite a wide range of fluorescent molecules or fluorophores, and a filter that blocks UV light can be placed in front of the camera lens so that only visible fluorescent light, which could have different colors, falls on the camera's detector array. A control/acquisition computer can be used to coordinate the illumination intensity with the camera's shutter in order to optimize the signal-to-noise of the detected fluorescent light.

Fluorescent or fluorescently labeled molecules that emit at different wavelengths can thus be imaged as different colors by the detecting camera, and time-lapse videos of the dynamics of reagent and product species can be detected in a manner similar to what we have demonstrated using optical absorption and white visible light illumination. Infrared (IR) wavelengths of light can also be used in illumination and/or detection; BCGE can utilize absorption, scattering, refraction, and fluorescence of IR light.

A potentially useful extension of BCGE involves labeling proteins and/or poly-nucleic acids (*e.g.* DNA and RNA) with different molecular fluorophores or fluorescent nanoparticles (*e.g.* quantum dots), and performing BCGE using UV (or visible) illumination to excite these fluorescent species while detecting the emitted fluorescent light in the visible (or IR) spectral range. Since bands of most proteins and poly-nucleic acids are typically invisible when illuminated with white visible light and since these biomolecules typically do not auto-fluoresce (with the exception being well-known proteins such as green fluorescent protein GFP and others of similar nature), it is typically necessary to fluorescently label such biomolecules in order to make them detectable and perform BCGE. It is typically desirable to label different biomolecules with different fluorophores, each of which emits light at a different wavelength, but all have excitation wavelengths in the range corresponding to the UV illumination. Once labeled, these biomolecules can be loaded into wells and BCGE can be performed using UV illumination and visible detection in the manner prescribed above. Alternatively, such biomolecules can be labeled subsequent to loading using BCGE by colliding a band of invisible biomolecules with a band of fluorescent molecules that bind to the biomolecules, such that the labeled biomolecules can be seen using UV-illumination BCGE. This extension of BCGE to a fluorescence imaging modality will provide access to a wide range of reactions of otherwise invisible species, such as

binding reactions, that include but are not limited to protein-protein, protein-DNA, protein-RNA, protein-ligand, DNA-DNA, and DNA-RNA reactions. By properly modulating the UV illumination intensity during the course of BCGE, the potential bleaching of fluorophores can be greatly reduced, and space-time plots of the reactions can be generated, just as we have demonstrated herein using visible white light illumination and absorbing dyes.

We envision that other experimental extensions of BCGE, related to optics of illumination and detection, could be readily implemented. In certain applications of BCGE, monochromatic or narrow-band polychromatic spectra of illumination could also be useful, rather than broadband illumination over wide continuous ranges of wavelengths. Likewise, the detection information can go beyond the limited and simple RGB detection offered by solid-state color array detectors. For instance, in the white light transmission geometry, by using 2D scanning spectroscopy detection, facilitated by a fiber optic detector that is connected to a digital spectrometer and pointed towards the gel region, wherein this fiber is scanned along the x-direction and also perpendicular to it in the plane above the gel using a computer-controlled mechanical x-y stage. A lens on the end of the fiber optic detector can collect light from a very small spatial region, in order to spatially resolve details in the local spectra of evolving flow patterns during BCGE. Such a detection would provide a spatially resolved intensity versus wavelength as a function of spatial position, similar to a RGB color array detector, but with full view of all spectroscopic details. Using fiber optic 2D scanning spectroscopy detection would overcome a well-known limitation of RGB imaging: limited three-channel RGB intensity data does not contain enough information in order to enable conversion into full spectral data of intensity versus wavelength. In some cases, it can be desirable for the electric field to be

temporarily turned off during the time period of 2D scanning of the fiber probe, so that the pattern of reagent and product species does not significantly evolve during this time period.

Programming Sequences of Collisions using BCGE

In the simplest and most direct implementation of BCGE, desired sequences of collisions of pulses of reagent species can be programmed by designing the relative spacing between wells in the same lane. Each well is loaded with only a single reagent species in a manner that provides the desired reaction sequence when the electric field is applied, given the electrophoretic mobilities of the reagent species. In addition to this implementation, for certain reaction sequences, one can also program a sequence of collisions of propagating pulses of reagent species using fewer wells in the same lane by an alternative method that takes advantage of differences in electrophoretic mobilities within the gel between two or more reagent species that are non-binding and non-reactive.

For example, in the same lane with only two wells, one can program a sequence of collisions involving separated pulses of anionic TZ and AR dye molecules that are initially loaded into a first well with a pulse of counter-propagating cationic MG that is initially loaded into a second well, separated from the first well by a distance of at least several centimeters. The TZ and AR dyes are both anionic, so they interact primarily by short-range screened electrostatic repulsion and do not exhibit any binding or reaction when loaded in the same well. Because these dyes have different electrophoretic mobilities, mostly as a consequence of -3*e* charge on TZ versus -2*e* charge on AR, when the electric field is applied, the TZ propagates more rapidly than AR in the gel, leading to two separate bands that sequentially collide with counter-propagating MG. The TZ-MG band-collision occurs first, and then any unreacted MG that continues propagating collides with the band of more slowly propagating AR.

Beyond propagation along only a single spatial direction, we envision that BCGE can be extended to involve programmable propagation and collision of reagent and product species along two or even three different orthogonal spatial directions. In a simple implementation, one or two different pairs of electrodes can be added to the pre-existing pair of electrodes in the basic one-dimensional BCGE apparatus. These sets of orthogonally situated electrodes can be used to create electric fields that cause propagation of reagent and/or product species involving components of velocities along both orthogonal Cartesian *x*- and *y*-directions in the plane of a slab-like gel or along *x*-, *y*-, and *z*-directions for thicker cube-like gels, respectively. For instance, in a two-dimensional version of BCGE, two orthogonal pairs of Pt electrodes would be mounted in four side-troughs below the gel region, rather than just one pair in two side troughs (as shown in Fig. 1a). The initial well locations for the reagent species would involve specifying both *x* and *y* coordinates in a slab-like gel, and it may be advantageous to stagger the loading wells rather than keep them in rows. The shapes of the wells may be made into circles or squares, rather than long rectangular bands, yielding packets of reagent species. Electric field components E_x and E_y can be varied independently and/or simultaneously in time through two different power supplies controlled by a single computer. Time-varying current (*e.g.* alternating current AC) rather than time-independent current (*e.g.* direct current DC) can further be used to drive each pair of electrodes, thereby providing a means of programming sequences of reactions using BCGE in 2D. Similar modifications can be made to adapt this extension of 2D-BCGE to 3D-BCGE.

Mitigating Gel-Matrix Interaction Effects in BCGE

When performing BCGE using certain reagent and product species, the potential effects of gel-matrix interactions on the reactions occurring within the gel can be largely neglected. This is typically true for small molecules and complexes in large-pore gels, and it is also typically true

for passivated gels that have surfaces to which reagent and product species do not bind. However, it can potentially be desirable to cause bands to collisionally react in spatial regions that are purposefully designed to be devoid of gel, thereby obviating gel-matrix effects that could potentially interfere with the reactions and formation of products that are not intrinsically bound to the gel. With this in mind, when casting the gel it is possible to design a void in addition to making the two wells into a lane of the gel at and around the location given by x^* where the band collision is predicted to occur. To appropriately fabricate the gel, it is necessary to know $\mu_{e,1}$ and $\mu_{e,2}$ in advance and to select L, the distance between wells, that enables a reasonable and physically accessible position of the void location at x^* to be made when casting the gel. Thus, a given lane would then have three regions devoid of gel; one each at $x = 0$ and $x = L$ corresponding to the wells, and in addition one at $x = x^*$ where the collision of bands occurs. If desired, once the collision occurs in the void, E can be eliminated, and the product species can be eluted or otherwise removed from the void region of the gel after a desired reaction time.

Supplementary Figures

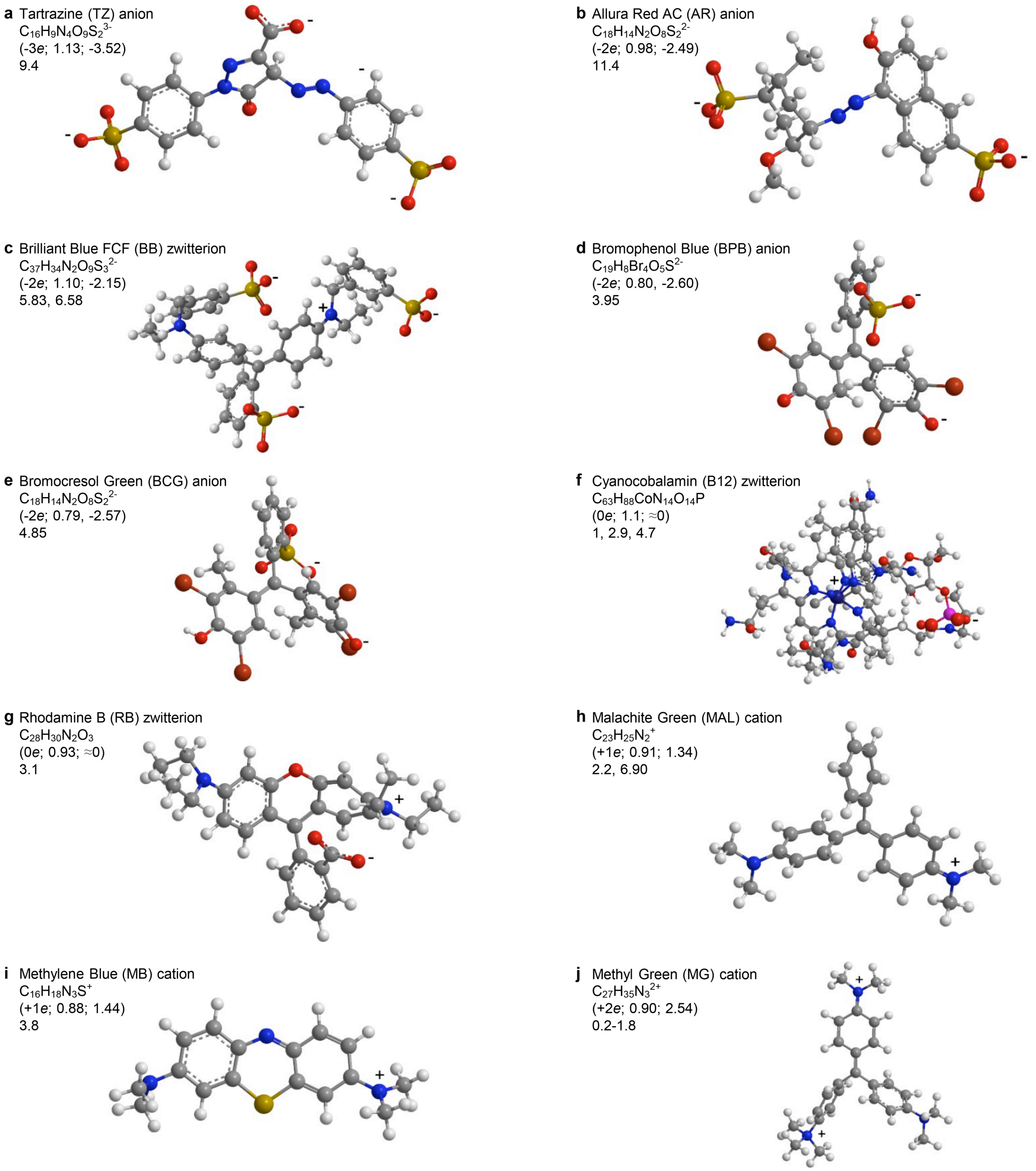

Supplementary Figure 1 Energy-optimized solvated configurations of organic dyes at pH = 9 based on minimized molecular models (HyperChem). **a**, Tartrazine (TZ) anion, 465.39 g mol^{-1}. **b**, Allura Red (AR) anion, 450.44 g mol^{-1}. **c**, Brilliant Blue (BB) zwitterion, 746.87 g mol^{-1}. **d**, Bromophenol Blue (BPB) anion, 667.95 g mol^{-1}. **e**, Bromocresol Green (BCG) anion, 698.02 g mol^{-1}. **f**, Cyanocobalamin (B12) zwitterion, 1,355.39 g mol^{-1}. **g**, Rhodamine B (RB) zwitterion, 442.56 g mol^{-1}. **h**, Malachite Green (MAL) cation, 329.47 g mol^{-1}. **i**, Methylene Blue (MB) cation, 284.40 g mol^{-1}. **j**, Methyl Green (MG) cation, 387.57 g mol^{-1}. Values in parentheses at the upper left of each panel provide for each molecule at pH = 9: predicted integer charge q (e); translational hydrodynamic radius a (in nm); and our measured value of electrophoretic mobility $\mu_{e,meas}$ (in 10^{-8} m^2 V^{-1} s^{-1}). Below this, we list reported pK_a value(s) or range(s) from literature sources; these are independent of pH. Charges are approximate and have been rounded to the nearest integer (see Supplementary Methods). Estimates of equivalent hydrodynamic sphere radii are made using WinHydroPro and HyperChem (see Methods).

Supplementary Tables

Compound	M_w (g mol^{-1})	Manufacturer	Product no.	CAS no.	Lot no.	Purity (manufacturer reported)
Tartrazine (as Acid Yellow 23) $C_{16}H_9N_4Na_3O_9S_2$	534.36	TCI	F0088	1934-21-0	W7BPB-HG	>98.0%
Allura Red AC $C_{18}H_{14}N_2Na_2O_8S_2$	496.42	TCI	A0943	25956-17-6	GJ01-QTAH	not provided
Brilliant Blue FCF (as Erioglaucine disodium salt, pure) $C_{37}H_{34}N_2Na_2O_9S_3$	792.84	ACROS Organics	229730050	3844-45-9	A0373695	"pure"
Bromophenol Blue (sodium salt) $C_{19}H_9Br_4NaO_5S$	691.94	Sigma Chemical Company	B6131	34725-61-6	16F-3675	technical grade
Bromocresol Green (0.1% w/v aqueous) $C_{21}H_{14}Br_4O_5S$	698.02	The Science Company	NC-13321	76-60-8	B10311241701	ACS reagent grade
Cyanocobalamin $C_{63}H_{88}CoN_{14}O_{14}P$	1355.37	Sigma Life Science	V2876	68-19-9	MKCB7713	≥98%
Rhodamine B (as Rhodamine 610 Chloride) $C_{28}H_{31}ClN_2O_3$	479.02	Exciton	Not provided	81-88-9	Lot # F	not provided
Malachite Green $C_{23}H_{25}ClN_2$	364.91	Matheson Coleman & Bell	B329	569-64-2	CMg 20 C.I. no. 42000	99%
Methylene Blue $C_{16}H_{18}ClN_3S$	319.85	Fisher	M-291	61-73-4	931790B	91%
Methyl Green $C_{27}H_{35}BrClN_3 \cdot ZnCl_2$	653.24	Sigma Chemical Company	No. M-8884	7114-03-6	65F-3657	90%
Heparin (sodium salt from porcine intestinal mucosa – Grade I-A)	203 USP units/mg	Sigma-Aldrich	H3393	9041-08-1	SLBG1344V	≥180 USP units/mg
Sodium dodecyl sulfate $NaC_{12}H_{25}SO_4$	288.37	MP Biomedical	811030	151-21-3	M9622	≥99%
Dodecyltrimethylammonium bromide $CH_3(CH_2)_{11}N(CH_3)_3Br$	308.35	Acros Organics	409311000	1119-94-4	A035872	99%
Hydrogen peroxide H_2O_2	34.01	Fisher Scientific	H327-500	7722-84-1	90592	34-37%
Potassium iodide KI	166.00	Alfa Aesar	11601	7681-11-0	P14C053	99.0% min
Strontium chloride SrCl	183.68	Mallinckrodt Chemical Works	N/A	10476-85-4	B-905	not provided
Sulfate-stabilized polystyrene spheres (*a* = 42 nm)	N/A	Interfacial Dynamics	1-80	9003-53-6 (polystyrene)	1524,1	N/A
Hydrochloric acid HCl (50% v/v)	36.46	Ricca	3580-16	10476-85-4	1606C62	50% (v/v)
Ethylenediaminetetraacetic acid $C_{10}H_{16}N_2O_8$	292.24	Acros Organics	446081000	60-00-4	A0379536	99%
Eriochrome black T $C_{20}H_{12}N_3O_7SNa$	461.38	Alfa Aesar	A17536	1787-61-7	10206882	not provided
Calcium chloride, dihydrate $CaCl_2 \cdot 2H_2O$	40.08	EMD	CX0130-1	10035-04-8	41046503	ACS reagent grade
Sodium borate, decahydrate $Na_2B_4O_7 \cdot 10H_2O$	381.42	Fisher Scientific	S25537A	1303-96-4	7GK37263	reagent grade
Chloro-acetic acid $ClCH_2COOH$	94.50	Alfa Aesar	A11482	79-11-8	W17A016	99%

Supplementary Table 1 Detailed compound information.

Fitting Parameters for Decomplexing of MG:BPB and MB:BB

For MG:BPB decomplexing (Fig. 6b), we fit measured time-dependent intensity profiles of the green channel using a modified semi-empirical Fermi-like function $I_{green} = I_b + I_{rise}/\{1 + \exp[-(t - \tau_0)/\tau_c]\}$. The fitting parameters are displayed in Supplementary Table 2. For MB:BB decomplexing (Fig. 6d), we fit the measured time-dependent intensity profiles to a semi-empirical log-normal function related to optical absorption of the stationary band of product: $I_{green} = 255 - \{A/[(t - \tau_i)\sigma]\} \exp[-\{\ln[(t - \tau_i)/\tau_d]\}^2/(2\sigma^2)]$ for $t > \tau_i$ and $I_{green} = 255$ for $0 \leq t \leq \tau_i$ (*i.e.* 255 corresponds to no optical absorption after ideal background subtraction). The fitting parameters are displayed in Supplementary Table 3.

$I_{green} = I_b + I_{rise}/\{1 + \exp[-(t - \tau_0)/\tau_c]\}$					
E_c (V/cm)	I_b	τ_0 (s)	τ_c (s)	I_{rise}	R^2
3.1	81.3 ± 0.5	1,876.2 ± 1.7	88.1 ± 1.5	166.2 ± 0.6	0.999
6.2	84.6 ± 0.9	932.5 ± 1.8	54.3 ± 1.5	166.8 ± 1.0	0.997
9.4	91.9 ± 1.3	665.0 ± 2.0	36.6 ± 1.8	154.1 ± 1.4	0.993

Supplementary Table 2 Fitting parameters for MG:BPB variable field strength experiments.

$I_{green} = 255 - \{A/[(t - \tau_i)\sigma]\} \exp[-\{\ln[(t - \tau_i)/\tau_d]\}^2/(2\sigma^2)]$ for $t > \tau_i$; $I_{green} = 255$ for $0 \leq t \leq \tau_i$					
E_c (V/cm)	A (s)	τ_i (s)	τ_d (s)	σ	R^2
3.1	$1.47 \pm 0.01 \times 10^5$	952.1 ± 4.7	$1.72 \pm 0.02 \times 10^3$	1.14 ± 0.01	0.983
6.2	$8.53 \pm 0.14 \times 10^4$	484.6 ± 5.3	881 ± 19	1.10 ± 0.02	0.969
9.4	$4.34 \pm 0.67 \times 10^4$	360.5 ± 2.3	454.2 ± 9.8	1.15 ± 0.02	0.984

Supplementary Table 3 Fitting parameters for MB:BB variable field strength experiments.

Supplementary References

1. Kiernan, J. A. Classification and naming of dyes, stains and fluorochromes. *Biotech. Histochem.* **76**, 261-278 (2001).

2. Guthrie, J. P. Hydrolysis of esters of oxy acids: pK_a values for strong acids; Brønsted relationship for attack of water at methyl; free energies of hydrolysis of esters of oxy acids; and a linear relationship between free energy of hydrolysis and pK_a holding over a range of 20 pK units. *Can. J. Chem.* **56**, 2342-2354 (1978).

3. Pérez-Urquiza, M. & Beltrán, J. L. Determination of the dissociation constants of sulfonated azo dyes by capillary zone electrophoresis and spectrophotometry methods. *J. Chromatogr. A* **917**, 331-336 (2001).

4. Xu, G. Simultaneous determination of food pigments in mixtures by pH fixed titration. *J. Shangqiu Teachers College* **15**, 79-83 (1999).

5. Flury, M. & Flühler, H. Brilliant blue FCF as a dye tracer for solute transport studies - A toxicological overview. *J. Environ. Qual.* **23**, 1108-1112 (1994).

6. Flury, M. & Flühler, H. Tracer characteristics of brilliant blue FCF. *Soil Sci. Soc. Am. J.* **59**, 22-27 (1995).

7. Patterson, G. S. A simplified method for finding the pKa of an acid-base indicator by spectrophotometry. *J. Chem. Ed.* **76**, 395-398 (1999).

8. Diamond, D., Lau, K. T., Brady, S. & Cleary, J. Integration of analytical measurements and wireless communications—Current issues and future strategies. *Talanta* **75**, 606-612 (2008).

9. Lexa, D. & Savéant, J.-M. Brönsted basicity of vitamin B12s. *J. Chem. Soc. Chem. Commun.* **0**, 872-874 (1975).

10. Arbeloa, I. L. & Ojeda, P. R. Molecular forms of rhodamine B. *Chem. Phys. Lett.* **79**, 347-350 (1981).

11. Iogannsen, M. G. Some structural features of vital dyes. *Bull. Exp. Bio. Med.* **83**, 591-595 (1977).

12. Goldacre, R. J. & Phillips, J. N. 370. The ionization of basic triphenylmethane dyes. *J. Chem. Soc.* 1724-1732 (1949).

13. Kim, J. R., Santiano, B., Kim, H. & Kan, E. Heterogeneous oxidation of methylene blue with surface-modified iron-amended activated carbon. *Am. J. Analyt. Chem.* **4**, 115-122 (2013).

14. Bishop, E. *Indicators*, 1st ed. (Oxford, Pergamon Press, 1972).

15. Ouyang, L., et al. Electronic structure and bonding in vitamin B-12, cyanocobalamin. *J. Mol. Struc.-Theochem.* **622**, 221-227 (2003).

16. Quast, R. Electroosmotic flow in agarose gels and value of agarose as stabilizing agent in gel electrofocusing. *J. Chromatogr.* **54**, 405-412 (1971).

17. Maitra, U., Mukhopadhyay, S., Sarkar, A., Rao, P. & Indi, S. S. Hydrophobic pockets in a nonpolymeric aqueous gel: Observation of such a gelation process by color change. *Angew. Chem. Int. Ed.* **40**, 2281-2283 (2001).

18. Ferreira, J. & Girotto, E. M. pH effects on the ohmic properties of bromophenol blue-doped polypyrrole film. *J. Brazil Chem. Soc.* **21**, 312-318 (2010).

19. Shokrollahi, A. & Firoozbakht, F. Determination of the acidity constants of neutral red and bromocresol green by solution scanometric method and comparison with spectrophotometric results. *Beni-Suef Univ. J. Appl. Sci.* **5**, 13-20 (2016).

20. Bell, S. ^{15}N NMR investigation of azo-hydrazone acid-base equilibria of FD and C yellow no. 5 (tartrazine) and two analogs. *Dyes Pigments* **11**, 93-99 (1989).

21. Shahir, A. A., Javadian, S., Razavizadeh, B. B. M. & Gharibi, H. Comprehensive study of tartrazine/cationic surfactant interaction. *J. Phys. Chem. B* **115**, 14435-14444 (2011).
