# Supplementary material for: Band-collision gel electrophoresis
Source: Nat Commun. 2019 Aug 12;10:3631. doi: 10.1038/s41467-019-11438-9 (PMC6690962; doi:10.1038/s41467-019-11438-9)
Supplement: Supplementary file 3 — Description of Additional Supplementary Files [file 41467_2019_11438_MOESM3_ESM.doc]

**Description of Additional Supplementary Files**

File Name: Supplementary Movie 1. Band-collision gel electrophoresis (BCGE) of oppositely charged dyes reveal complex formation.

Description: 4.5 mM bands of anionic TZ, AR, and BB collide with cationic MAL and MB (Fig. 2). 5.0 mM SBB at pH = 9.0 in a 3.0% (w/w) agarose gel at an electric field strength *E* = 3.1 V cm^-1^ (see Fig. 1a). Anionic dyes propagate upward; cationic dyes propagate downward. Scale: wells measure 6.5 mm wide for this movie only. Acquisition duration: 3,585 s (*i*.*e*. 240 frames at 1/15 frames per second (FPS)). Movie playback: duration 16.0 s at 15 FPS.

File Name: Supplementary Movie 2. Electrophoresis of organic dye molecules yield measured mobilities.

Description: Bands of organic dyes (see Fig. 2) propagate in a 3.0% (w/w) agarose gel in 5.0 mM SBB at pH = 9.0 at an electric field strength *E* = 3.1 V cm^-1^. Left to right, dyes are TZ, AR, BB, BPB, BCG, B12, RB, MAL, MB, and MG (see Fig. 3a). Scale: dashed-line wells are 4 mm wide for this movie and subsequent movies. Acquisition duration: 2,545 s (*i.e.* 170 frames at 1/15 FPS). Movie playback: duration 11.3 s at 15 FPS.

File Name: Supplementary Movie 3. Bands of B12 and AR(-2*e*) collide and pass through each other without interacting.

Description: 4.5 mM bands of near-neutral B12 and anionic AR collide (Fig. 4a). Conditions: same as Supplementary Movie 2. Acquisition duration: 5,100 s (*i.e.* 341 frames at 1/15 FPS). Movie playback: duration 22.7 s at 15 FPS.

File Name: Supplementary Movie 4. BCGE of equimolar TZ(-3*e*) and MB(+*e*) yields anionically charged complexes.

Description: 4.5 mM bands of anionic TZ and cationic MB collide, demonstrating strong attractive interaction and complex formation without neutralization (Fig. 4d). Conditions: same as Supplementary Movie 2. Acquisition duration: 3,960 s (*i.e.* 265 frames at 1/15 FPS). Movie playback: duration 17.7 s at 15 FPS.

File Name: Supplementary Movie 5. BCGE of TZ(-3*e*) and MB(+*e*) at different relative concentrations. The higher charge and lower stoichiometric ratio of TZ:MB (ranging from 5:1 to 1:5) is apparent at high relative concentrations of MB to TZ for which TZ is noticeably slowed (Fig. 5a). Conditions: same as Supplementary Movie 2. Acquisition duration: 3,600 s (*i.e.* 241 frames at 1/15 FPS). Movie playback: duration 16.1 s at 15 FPS.

File Name: Supplementary Movie 6. BCGE of AR(-2*e*) and MG(+2*e*) at different relative concentrations.

Description: For the 1:1 concentration [AR]:[MB], the complex resulting from collision remains relatively stationary, whereas for 5:1 and 1:5, complexes propagate upwards or downwards to a similar extent, respectively. Conditions: same as Supplementary Movie 2. Acquisition duration: 4,065 s (*i*.*e*. 272 frames at 1/15 FPS). Movie playback: duration 18.1 s at 15 FPS.

File Name: Supplementary Movie 7. BCGE of BPB(-2*e*) and MG(+2*e*) yields neutral stationary complexes.

Description: The complexes formed in a narrow band are clearly stationary, implying neutrality. Subsequent decomplexing leads to a butterfly pattern, consistent with a 1:1 stoichiometric ratio of BPB : MG (Fig. 4g). Conditions: same as Supplementary Movie 2. Acquisition duration: 5,280 s (*i.e.* 353 frames at 1/15 FPS). Movie playback: duration 11.8 s at 30 FPS.

File Name: Supplementary Movie 8. BCGE of BB(-2*e*) and MB(+*e*) yields a stationary band of a longer-lived insoluble complex-precipitate.

Description: The band of complex-precipitate formed upon collision is stationary and only very slowly decomplexes and dissolves, causing a high degree of smearing of bands. Based on differences in the optical absorption as the decomplexing is occurring, the complex appears to be composed of predominantly MB (Fig. 4j). Conditions: same as Supplementary Movie 2. Acquisition duration: 5,925 s (*i.e.* 396 frames at 1/15 FPS). Movie playback: duration 6.6 s at 60 FPS.

File Name: Supplementary Movie 9. BCGE of singly protonated BPB(-*e*) and MG(+2*e*) in acidic buffer.

Description: BPB(-2*e*, blue at pH = 9), when placed in 5.0 mM chloro-acetic acid (CAA) buffer at pH = 2.87, becomes singly protonated to BPB(-*e*, yellow), while MG remains in a +2*e* charge state. Collision yields a stationary band of complex that only slowly decomplexes/dissolves (Fig. 7a). Conditions: 3.0% (w/w) agarose, *E* = 3.1 V cm^-1^. Acquisition duration: 9,090 s (*i*.*e*. 607 frames at 1/15 FPS). Movie playback: duration 40.5 s at 15 FPS.

File Name: Supplementary Movie 10. BCGE of BPB(-2*e*) and H_3_O^+^: transient protonation and ejected plumes.

Description: Bands of BPB change color from blue to yellow and back to blue after colliding with invisible counter-propagating bands of H_3_O^+^ at 1.0, 0.5, and 0.25 M initial concentrations. A more rapidly-propagating plume of BPB is ejected from the leading edges of the bands (Fig. 7b and 7c). Conditions: same as Supplementary Movie 2. Acquisition duration: 1,350 s (*i.e.* 91 frames at 1/15 FPS). Movie playback: duration 6.1 s at 15 FPS.

File Name: Supplementary Movie 11. BCGE of invisible poly-anionic heparin (HEP) and MB(+*e*).

Description: Complexes having different stoichiometry and propagation rates are formed; the degree of HEP:MB complexing is indicated by a change from blue to purple color (Fig. 7h). Levels have been adjusted to provide increased visibility of decomplexing MB propagating downwards. Conditions: same as Supplementary Movie 2. Acquisition duration: 14,370 s (*i*.*e*. 959 frames at 1/15 FPS). Movie playback: duration 16.0 s at 60 FPS.

File Name: Supplementary Movie 12. BCGE of invisible surfactant DS^-^ and dyes B12, RB, MAL, MB, and MG.
Description: Bands of DS^-^ anions pass through bands of neutral B12 and RB with no observable interaction, whereas DS^-^ forms complexes having different longevities with cationic MAL, MB, and MG. Conditions: same as Supplementary Movie 2. Acquisition duration: 4,935 s (*i*.*e*. 330 frames at 1/15 FPS). Movie playback: duration 22.0 s at 15 FPS.

File Name: Supplementary Movie 13. BCGE of invisible surfactant DTA^+^ and anionic dyes TZ, AR, BB, BPB, and BCG.
Description: Complexes of DTA^+^ with these anionic dyes form upon collision, yielding stationary bands that can be seen most easily for AR, BPB, and BCG. Conditions: same as Supplementary Movie 2. Acquisition duration: 4,815 s (*i.e.* 322 frames at 1/15 FPS). Movie playback: duration 21.5 s at 15 FPS.
