# Supplementary material for: Band-collision gel electrophoresis
Source: Nat Commun. 2019 Aug 12;10:3631. doi: 10.1038/s41467-019-11438-9 (PMC6690962; doi:10.1038/s41467-019-11438-9)
Supplement: Supplementary file 2 — Peer Review [file 41467_2019_11438_MOESM2_ESM.pdf]

Reviewers' comments:

Reviewer #1 (Remarks to the Author):

The authors demonstrate a new way of visualising the effective mobility of interacting molecules. The method is similar to electrophoretic mobility shift assays (EMSA), but is dynamic. Typically, a pulse of positively charged dye is collided with a pulse negatively charged dye in a gel. Any interaction between the molecules is clear through a change in migration velocity. I am not a true expert in electrophoretic methods or EMSA, but thoroughly enjoyed the work and believe it is novel and would be of interest to a wider community.
It feels a bit like a curiosity, where the impact is not clear. It may influence thinking in the field because they provide a new way to visualise and think about reacting species in a dynamic system. It is a very satisfying read, in no small part because of the excellent writing and figures.
I would recommend this for publication after considering some minor comments detailed below.

Sincerely
David Inglis

a) Somewhere around line 97, I was looking for the authors to estimate or measure the effective mobility of a complex. Why wasn't this done? Is it just too hard to get a good number of hydrodynamic size? I again hoped for some details on the complexes effective mobility at line 137.
b) Line 119: add color tags: TZ(-3e, yellow) and MB(+e, Blue) to make understanding the figure easier.
c) Line 200: Can you speculate on the fate of the EDTA-CA complex? Where does it go?
d) line 276: Perhaps this is a naive question, but why was D2O used?
e) Line 335, Table 1: more detail of what the numbers on the bottom of each box mean. The first is charge, is the second one hydrodynamic radius in nm?, what is the the third? is the fourth the effective mobility?

f) Line 336, Figure 1: figure axis labels should be increased in size.

g) Figures in general. Why not embed the figures in the text where they are called? To review this paper, I need to have two copies of the pdf open on my computer to view the figure and the relevant text at the same time. Placing the figures at the end of the manuscript was a sensible thing in the era of typewriters.

Reviewer #2 (Remarks to the Author):

Article: NCOMMS-19-01942-T
Title: Band-collision gel electrophoresis
Authors: Dimitri A. Bikos and Thomas G. Mason

General comments

This is a novel study, using an interesting variant of the electrophoretic mobility shift assay. The text is brief, clear and well written. I recommend acceptance following revision as described below.

The samples consist of several small, organic dye molecules, of differing charge. The use of two starting zones allows observation of collisional interactions, and an estimation of rate complexes for formation and dissociation of complexes. Effects of field strength (hence interaction time of colliding bands), pulses of acid (as one of the reactants), and ionic complex exchange reactions are probed. The time-dependent mobility measurements and space-time analyses are strengths of the work.

However, important elements are missing from this presentation.

1. Several of the dyes tested have been in use for decades, and it seems likely that interaction studies using other techniques (e.g., spectrophotometry) have been performed, on at least a few. This feature is not discussed at all in the current manuscript and the observed interactions are presented without scientific or historical context.

2. Reaction kinetics have been measured by EMSA, for several molecular systems. The authors do not discus these precedents. Examples are given in Specific Comments below, and this list is by no means comprehensive. As the current assay has several novel features, its historical and technical context should be discussed.

3a. Reaction kinetics have been measured within EMSA gel matrices before (refs 4 and 9 below for example), yet the current work does not discuss this historical and technical context.

3b. Important gel-matrix effects were observed in previous EMSA studies, so it would be worth the author's time to comment on why they do not think the gel matrix exerts comparable effects their system. Do they have evidence from use of chemically-different matrices, or matrices with different pore sizes?

4. Matrix-isolation methods have been in use for studies of reactive molecules for decades. See for instance:

Eric Whittle, David A. Dows, George C. Pimentel (1954). "Matrix Isolation Method for the Experimental Study of Unstable Species". The Journal of Chemical Physics. 22 (11): 1943

Riedel, Sebastian; Köchner, Tobias; Wang, Xuefeng; Andrews, Lester (2 August 2010). "Polyfluoride Anions, a Matrix-Isolation and Quantum-Chemical Investigation". Inorganic Chemistry. 49 (15): 7156–7164.

The authors should give some discussion of the historical context of this feature of their experimental method.

Specific Comments

L.33: "EMSAs are typically used to probe equilibrium biomolecular binding…" While equilibrium EMSAs are most widely used, there is a considerable literature describing kinetic measurements made by EMSA. See the following references(1-9).

1. Fried, M.G. and Crothers, D.M. (1981) Equilibria and Kinetics of Lac Repressor-Operator Interactions by Polyacrylamide Gel Electrophoresis. Nucl. Acids Res., 9, 6505-6525.
2. Fried, M.G. and Crothers, D.M. (1984) Kinetics and mechanism in the reactions of gene regulatory proteins with DNA. J. Mol. Biol., 172, 263-282
3. Ruusala, T. and Crothers, D.M. (1992) Sliding and intermolecular transfer of the lac repressor: kinetic perturbation of a reaction intermediate by a distant DNA sequence. Proc Natl Acad Sci U S A., 89, 4903-4907.
4. Fried, M.G. and Liu, G. (1994) Molecular Sequestration Stabilizes CAP-DNA Complexes During Polyacrylamide Gel Electrophoresis. Nucl. Acids Res., 22, 5054-5059.
5. Bellamy, S.R., Kovacheva, Y.S., Zulkipli, I.H. and Halford, S.E. (2009) Differences between Ca2+ and Mg2+ in DNA binding and release by the SfiI restriction endonuclease: implications for DNA looping. Nucleic Acids Res., 37, 5443-5453.
6. Sidorova, N.Y., Muradymov, S. and Rau, D.C. (2011) Solution parameters modulating DNA binding specificity of the restriction endonuclease EcoRV. FEBS J., 278, 2713-2727.

7. Goodrich, J.A. and Kugel, J.F. (2015) Studying the affinity, kinetic stability, and specificity of RNA/protein interactions: SINE ncRNA/Pol II complexes as a model system. Methods Mol Biol., 1206, 165-178.
8. Fried, M.G. and Bromberg, J.L. (1997) Factors that affect the stability of protein-DNA complexes during gel electrophoresis. Electrophoresis, 18, 6-11.
9. Vossen, K.M. and Fried, M.G. (1997) Sequestration Stabilizes Lac Repressor-DNA Complexes During Gel Electrophoresis. Anal. Biochem., 245, 85-92.

L.78: The dye molecules tested are much smaller than the gel pores. This condition does not apply for molecular systems containing proteins or typically-sized nucleic acids.

L.87: The use of continuous monitoring of band mobility and space-time plots are very nice features of this work.

Reviewer #3 (Remarks to the Author):

This paper by Bikos & Mason presents a clever and interesting adaptation of gel electrophoresis to detect molecular interactions. The approach, for which they have coined the term band-collision electrophoresis (BCGE), involves electrophoresing oppositely-charged species from different starting points in an agarose gel, oriented such that the bands of reacting species migrate towards one another. The bands either pass through one another or if they associate, form complex(es) whose charge(s) and therefore electrophoretic mobilities differ from the reacting species. This yields bands that migrate at different rates, remain stationary or even reverse direction. Subsequently, as the complexes dissociate under thermal fluctuations the reactants of opposite charge separate causing the complex band to dissolve into smears of various sorts.

The idea is that the particular patterns obtained are indicative of the mobilities of the reacting species, affinity of interactions and the charge, including coupled acid-base chemistry, stoichiometry and kinetics of complex formation/dissociation, factors that might be influenced or assessed by varying the concentrations of reacting species, pH of the gel environment and electric field strength. The authors demonstrate these effects using small molecule dyes with absorption in the visible range for easy, separate detection.

Several limitations to this approach in application are evident, e.g., requirement for reactants of opposite charge. The reactant bands present concentration gradients in the leading edges where association occurs, and while the authors are correct that this offers additional potential information about affinity and kinetics of complex formation they are also note correctly that predicting the evolving patterns presents a daunting challenge for modeling, simulation and micro spatial detection. Finally, a logical limitation is that while a number of distinct patterns are demonstrated, several of these resulting from different interactions are quite similar. While it may be possible to go from interaction to pattern, it is not evident that it will be possible to go in the reverse direction uniquely. Consequently, it isn't clear how this will be useful to address any of the individual variables.

The introduction references GE used for analysis of biological macromolecules, i.e., nucleic acids and proteins in SDS, and gel-mobility shift assays for protein-DNA interactions. These interactions add complexity due to the lower electrophoretic mobility of the various species overall, uncertain charge differences, heterogeneity of interacting species and in complexes formed, overall size of the species and caging effects of the gel matrix, etc. While BCGE certainly could be applied to protein-DNA interactions in many cases, depending on the charge of the protein species, the advantages over standard gel-shift GE aren’t immediately obvious.

In conclusion, this presents a very nice demonstration in principle of a clever approach that should motivate its further development. The additional work necessary to develop useful analytical techniques is significant.

Reviewer #1 (David Inglis):

The authors demonstrate a new way of visualising the effective mobility of interacting molecules. The method is similar to electrophoretic mobility shift assays (EMSA), but is dynamic. Typically, a pulse of positively charged dye is collided with a pulse negatively charged dye in a gel. Any interaction between the molecules is clear through a change in migration velocity. I am not a true expert in electrophoretic methods or EMSA, but thoroughly enjoyed the work and believe it is novel and would be of interest to a wider community.

> We thank Prof. Inglis for these positive comments about our work, particularly about its novelty.

It feels a bit like a curiosity, where the impact is not clear. It may influence thinking in the field because they provide a new way to visualise and think about reacting species in a dynamic system. It is a very satisfying read, in no small part because of the excellent writing and figures.

> We agree that real-time visualization is a very appealing aspect of our approach, and we appreciate these positive remarks about our writing and figures.

I would recommend this for publication after considering some minor comments detailed below.

> We thank Prof. Inglis for recommending publication, and we have improved our manuscript based on his detailed comments below.

a) *Somewhere around line 97, I was looking for the authors to estimate or measure the effective mobility of a complex. Why wasn't this done? Is it just too hard to get a good number of hydrodynamic size? I again hoped for some details on the complexes effective mobility at line 137.*

> This is a very good point, and we now provide measured values of the effective electrophoretic mobilities of complexes at the two points in the manuscript that are mentioned here. These mobilities are measured values and do not rely upon any estimate of hydrodynamic size of these complexes, which could in principle be obtained through modeling that is significantly more complicated than what we have performed for single dye molecules.

b) *Line 119: add color tags: TZ(-3e, yellow) and MB(+e, Blue) to make understanding the figure easier.*

> We have added these color tags to improve the clarity of this figure.

c) *Line 200: Can you speculate on the fate of the EDTA-CA complex? Where does it go?*

> Yes, we can. EDTA-Ca complexes will have net negative charge because of the excess negative charge in the $EDTA^{4-}$ compared to Ca^{2+}. The chelation capacity of EDTA is greatly reduced when one Ca^{2+} is bound, so the likelihood of neutral EDTA-2Ca complexes being formed and remaining stable is extremely low. While we don't visualize the invisible EDTA-Ca complex, it would migrate as a negatively charged complex. We now briefly mention this in our revised manuscript.

d) line 276: Perhaps this is a naive question, but why was D_2O used?

> We follow a standard well-loading method in gel electrophoresis, by adding D_2O to the dye solution. D_2O has a higher mass density than H_2O and therefore causes the dye molecules to sink to the bottom of the wells, prior to turning on the electric field. This provides better uniformity in the vertical location of the loaded dyes in the wells prior to migration and collision. We now note this explicitly in the Methods section.

e) Line 335, Table 1: more detail of what the numbers on the bottom of each box mean. The first is charge, is the second one hydrodynamic radius in nm?, what is the the third? is the fourth the effective mobility?

> We thank Prof. Inglis for pointing out this omission in the legend of Table 1. We now define the meaning of these numbers and provide their units in the legend (see bottom of Table 1).

f) Line 336, Figure 1: figure axis labels should be increased in size.

> In our revised manuscript, we have increased the size of the labels on the figure axes of Figure 1 in our original manuscript, which is now Figure 2 of our revised manuscript.

g) Figures in general. Why not embed the figures in the text where they are called? To review this paper, I need to have two copies of the pdf open on my computer to view the figure and the relevant text at the same time. Placing the figures at the end of the manuscript was a sensible thing in the era of typewriters.

> In our revised manuscript for review-only purposes (in pdf format), we have embedded all Figures and the Table, as well as their legends, in the text at the appropriate places near where they are called. We hope that this facilitates review of our revised submission. However, in the primary Word file of our resubmitted manuscript, per guidelines of *Nature Communications*, we have omitted the Figures (which have been supplied as separate editable graphics files) and have grouped all figure legends near the end of the document.

Reviewer #2:
This is a novel study, using an interesting variant of the electrophoretic mobility shift assay. The text is brief, clear and well written. I recommend acceptance following revision as described below.

> We thank Reviewer #2 for these very positive comments about the novelty of our experimental approach and the clarity of our writing and for recommending acceptance following revisions, which we are certainly happy to make.

The samples consist of several small, organic dye molecules, of differing charge. The use of two starting zones allows observation of collisional interactions, and an estimation of rate complexes for formation and dissociation of complexes. Effects of field strength (hence interaction time of colliding bands), pulses of acid (as one of the reactants), and ionic complex exchange reactions are probed. The time-dependent mobility measurements and space-time analyses are strengths of the work.

> We thank Reviewer #2 for these positive remarks about our time-dependent mobility measurements and space-time analyses.

However, important elements are missing from this presentation.

1. *Several of the dyes tested have been in use for decades, and it seems likely that interaction studies using other techniques (e.g., spectrophotometry) have been performed, on at least a few. This feature is not discussed at all in the current manuscript and the observed interactions are presented without scientific or historical context.*

> Reviewer #2 is correct in pointing out that studies have been performed decades ago using absorption spectrophotometry on mixtures of two different dye solutions, including both anionic and cationic dye species, in order to discern interactions between unlike dye molecules. In our introduction, we now point this out, providing a scientific and historical context surrounding the primary examples of dyes used in our experiments, and we now cite appropriate references. While we have attempted to find literature that contains spectrophotometry data on mixtures of the exact pairs of dyes that we have used in our BCGE experiments, we have not found precise matches. Nevertheless, the reader now has clear links to the literature regarding non-additive absorption spectra of the mixtures of strongly interacting dye molecules that are in similar classes of dyes as the ones we have used for BCGE. We thank Reviewer #2 for this constructive comment, which has led to a significant improvement to our introduction.

2. *Reaction kinetics have been measured by EMSA, for several molecular systems. The authors do not discus these precedents. Examples are given in Specific Comments below, and this list is by no means comprehensive. As the current assay has several novel features, its historical and technical context should be discussed.*

> We agree that our highly abbreviated presentation of the historical and technical context regarding EMSAs could have been better, so in our revised manuscript we have significantly expanded our introduction related to EMSAs, also taking into account the Specific Comments and references below. We believe that the introduction in our revised manuscript has been significantly improved, and the historical and

technical context for our innovations can be better appreciated by the reader. We address the Specific Comments in more detail below.

3a. Reaction kinetics have been measured within EMSA gel matrices before (refs 4 and 9 below for example), yet the current work does not discuss this historical and technical context.

> In the introduction of our revised manuscript, we now discuss the use of EMSA studies for revealing kinetics, not just equilibrium binding properties, including observable differences in kinetics within EMSA gel matrices; we also cite the appropriate references that Reviewer #2 has given here.

3b. Important gel-matrix effects were observed in previous EMSA studies, so it would be worth the author's time to comment on why they do not think the gel matrix exerts comparable effects their system. Do they have evidence from use of chemically-different matrices, or matrices with different pore sizes?

> Gel matrix effects can be important in BCGE, and this is evident particularly in the absence of propagation of certain products that are formed through aggregation or precipitation into much larger objects that exceed the characteristic pore size of the gel. This is particularly evident in Fig. 6j and 6k, which shows the irreversible BCGE-reaction involving Sr^{2+} and anionic nanospheres in a PEG-passivated large-pore agarose gel. We comment on the characteristic pore size in more detail below (related to L.78). Certainly PEG-passivation is necessary to enable propagation of anionic nanospheres in agarose gels, and we have cited appropriate literature related to this in the Methods of our original submission. In the Discussion of our revised submission, we also point out in future directions that exploring the interactions between the gel-matrix, reactant species, and product species as well as the characteristic pore size of the gel, would be interesting to explore.

4. Matrix-isolation methods have been in use for studies of reactive molecules for decades. See for instance:

Eric Whittle, David A. Dows, George C. Pimentel (1954). "Matrix Isolation Method for the Experimental Study of Unstable Species". The Journal of Chemical Physics. 22 (11): 1943

Riedel, Sebastian; Köchner, Tobias; Wang, Xuefeng; Andrews, Lester (2 August 2010). "Polyfluoride Anions, a Matrix-Isolation and Quantum-Chemical Investigation". Inorganic Chemistry. 49 (15): 7156–7164.

The authors should give some discussion of the historical context of this feature of their experimental method.

> In the introduction of our revised manuscript, we now mention matrix-isolation methods for studying reactive molecules and cite these references.

Specific Comments

L.33: "EMSAs are typically used to probe equilibrium biomolecular binding…" While equilibrium EMSAs are most widely used, there is a considerable literature describing kinetic measurements made by EMSA. See the following references(1-9).

1. *Fried, M.G. and Crothers, D.M. (1981) Equilibria and Kinetics of Lac Repressor-Operator Interactions by Polyacrylamide Gel Electrophoresis. Nucl. Acids Res., 9, 6505-6525.*
2. *Fried, M.G. and Crothers, D.M. (1984) Kinetics and mechanism in the reactions of gene regulatory proteins with DNA. J. Mol. Biol., 172, 263-282*
3. *Ruusala, T. and Crothers, D.M. (1992) Sliding and intermolecular transfer of the lac repressor: kinetic perturbation of a reaction intermediate by a distant DNA sequence. Proc Natl Acad Sci U S A., 89, 4903-4907.*
4. *Fried, M.G. and Liu, G. (1994) Molecular Sequestration Stabilizes CAP-DNA Complexes During Polyacrylamide Gel Electrophoresis. Nucl. Acids Res., 22, 5054-5059.*
5. *Bellamy, S.R., Kovacheva, Y.S., Zulkipli, I.H. and Halford, S.E. (2009) Differences between Ca2+ and Mg2+ in DNA binding and release by the SfiI restriction endonuclease: implications for DNA looping. Nucleic Acids Res., 37, 5443-5453.*
6. *Sidorova, N.Y., Muradymov, S. and Rau, D.C. (2011) Solution parameters modulating DNA binding specificity of the restriction endonuclease EcoRV. FEBS J., 278, 2713-2727.*
7. *Goodrich, J.A. and Kugel, J.F. (2015) Studying the affinity, kinetic stability, and specificity of RNA/protein interactions: SINE ncRNA/Pol II complexes as a model system. Methods Mol Biol., 1206, 165-178.*
8. *Fried, M.G. and Bromberg, J.L. (1997) Factors that affect the stability of protein-DNA complexes during gel electrophoresis. Electrophoresis, 18, 6-11.*
9. *Vossen, K.M. and Fried, M.G. (1997) Sequestration Stabilizes Lac Repressor-DNA Complexes During Gel Electrophoresis. Anal. Biochem., 245, 85-92.*

> We thank Reviewer #2 for pointing out the significant prior work regarding the use of EMSAs for revealing kinetics. In our revised introduction, we now cite this prior work and ensure that readers have a more complete picture about how EMSAs have been used to study kinetics in addition to equilibrium binding.

L.78: The dye molecules tested are much smaller than the gel pores. This condition does not apply for molecular systems containing proteins or typically-sized nucleic acids.

> This is a very good point. Most of our experiments involve reagents (e.g. dye molecules) and products (e.g. complexes) that are much smaller than the characteristic pore size (≈ 50 nm) of the agarose gel at the concentration we used. However, we have performed reactions involving much larger charged nanoparticle reagents (see Figure 6j and 6k of our revised submission). Although individual nanoparticles can propagate as a coherent band through the gel initially, when this band collides with a band of Sr^{2+} binding counterions, these nanoparticles irreversibly form large aggregates compared to the gel's pore size, thereby inhibiting further propagation. In our revised Discussion, we now say that it would be interesting to explore BCGE more comprehensively through studies of biomacromolecules, including proteins and poly-nucleic acids. The behavior of these in different types and concentrations of gels, leading to different sizes of reagent and product species relative to the pores and struts of the gel and different interactions of these species with the gel matrix (or even how the gel might interfere to a certain degree with binding) would be very interesting to study.

L.87: The use of continuous monitoring of band mobility and space-time plots are very nice features of this work.

> We thank Reviewer #2 for this positive comment about continuously monitoring band mobility and about our use of space-time plots.

Reviewer #3:

This paper by Bikos & Mason presents a clever and interesting adaptation of gel electrophoresis to detect molecular interactions. The approach, for which they have coined the term band-collision electrophoresis (BCGE), involves electrophoresing oppositely-charged species from different starting points in an agarose gel, oriented such that the bands of reacting species migrate towards one another. The bands either pass through one another or if they associate, form complex(es) whose charge(s) and therefore electrophoretic mobilities differ from the reacting species. This yields bands that migrate at different rates, remain stationary or even reverse direction. Subsequently, as the complexes dissociate under thermal fluctuations the reactants of opposite charge separate causing the complex band to dissolve into smears of various sorts.

> We thank Reviewer #3 for such positive comments, including calling our work "a clever and interesting adaptation of gel electrophoresis".

The idea is that the particular patterns obtained are indicative of the mobilities of the reacting species, affinity of interactions and the charge, including coupled acid-base chemistry, stoichiometry and kinetics of complex formation/dissociation, factors that might be influenced or assessed by varying the concentrations of reacting species, pH of the gel environment and electric field strength.

> We thank Reviewer #3 for nicely summarizing many of the reaction types and GE conditions that we have demonstrated in our initial presentation of BCGE.

The authors demonstrate these effects using small molecule dyes with absorption in the visible range for easy, separate detection.

> While it is true that most of our examples involved small dye molecules that absorb in the visible range, BCGE is not limited only to such molecules. Beyond dye molecules, our reagents included charged nanospheres that scatter visible light and effectively non-absorbing (*i.e.* 'invisible') molecules, such as ionic surfactants and heparin.

Several limitations to this approach in application are evident, e.g., requirement for reactants of opposite charge.

> We thank Reviewer #3 for this comment, which is essentially addressing potential limitations of the BCGE method. Although many of our examples involve the counter-propagating band-collision scenario, in fact, there is no strict requirement that the reactants must have opposite charge. In order to provide readers with a comprehensive view of the experimental apparatus and of possible collision scenarios that BCGE can achieve, we have composed and included a new schematic Fig. 1 in our revised submission. This new Fig. 1 clearly explains the possible collision scenarios in BCGE graphically. We have also provided a solution to simultaneous equations in position for

the centers of the bands, leading to equations for both the collision time t^* and the collision location x^*. We believe that this new figure and these new equations will further enhance the reader's appreciation of the generality of BCGE.

Nevertheless, we appreciate the spirit of this comment. The range of reagent species that can be used in BCGE does have practical limitations, and these need to be better specified. We now address this directly in our revised Introduction and Methods. Specifically, the BCGE method is limited when the electrophoretic mobilities of the reactants have the same sign and are close enough in magnitude that obtaining a collision location within a finite-size gel, which fits into the GE apparatus, becomes practically infeasible.

As we have shown in our initial submission, we can use BCGE to react molecules or supramolecular objects that have a sufficiently large difference in electrophoretic mobilities, not merely electrophoretic mobilities μ_e that have opposite signs (i.e. reactants of opposite charge). In our original submission, we also demonstrated a redox reaction involving neutral hydrogen peroxide (zero charge) and negatively charged iodide (see Fig. 5 l of our original submission, or Fig. 6l of our revised submission).

Although not shown in any examples in our initial submission, in other BCGE experiments that we have performed, we have also collided two different negatively charged dyes (*e.g.* AR and BB) that have the same sign but different magnitudes of μ_e. Although these dyes did not give any evidence of noticeable attractive interactions, since the band of AR propagated through the band of BB without disturbance of either, likely because of short-range charge repulsion, the collision of these bands was nevertheless achieved. For other types of reactions, such as redox, the collision of same-sign reagent species may still yield reaction products; this has yet to be explored.

The reactant bands present concentration gradients in the leading edges where association occurs, and while the authors are correct that this offers additional potential information about affinity and kinetics of complex formation they are also note correctly that predicting the evolving patterns presents a daunting challenge for modeling, simulation and micro spatial detection.

> We thank Reviewer #3 for saying that we have correctly identified additional potential information in the patterns generated by BCGE as well as the daunting challenge ahead for modeling, simulation, and micro-spatial detection. While daunting, we see this challenge as a positive aspect that may motivate future progress in many different directions. One can imagine including the appropriate and essential ingredients about diffusion, porous media, electrophoretic propagation, and reactions in a model or simulation that could, at least in concept, yield patterns that would be comparable to those we have observed. In chemical engineering, finite-element simulations that include convection-diffusion-reaction scenarios have been addressed, typically without including the porous medium; it may be possible to further modify these simulations to incorporate the porous medium and interactions of species with this medium. Regarding micro-spatial detection, we have enhanced our Supplementary Discussion,

outlining a reasonable path to an optical fiber scanning methodology that could potentially be used in future experiments to obtain spatially resolved spectrophotometric data, rather than RGB data, through BCGE.

Finally, a logical limitation is that while a number of distinct patterns are demonstrated, several of these resulting from different interactions are quite similar. While it may be possible to go from interaction to pattern, it is not evident that it will be possible to go in the reverse direction uniquely. Consequently, it isn't clear how this will be useful to address any of the individual variables.

> We see BCGE as a versatile empirical method that provides a parallelized and programmable method of controlled reactions of small quantities of reagent species, suitable for a wide range of chemical reactions. Assessing the uniqueness of inverse problems for different reagents and reaction types is an interesting subject for future work. We believe that individual variables can be addressed, perhaps only in some cases even if not all. While we agree with Reviewer #3 that the uniqueness of inverse solutions in going from the pattern generated by BCGE back to fundamental variables over the full range of possible reagents and scenarios is unresolved at this point. We mention this now in our revised Discussion.

By analogy, just because the famous phase problem existed in scattering experiments from the very earliest stages, the existence of this phase problem did not preclude the obvious utility of this technique in obtaining very useful scattering intensity information as a function of wavevector, which typically requires modeling to interpret in terms of real-space structure. The absence of phase information, which would have made the direct solution of the real-space structure possible to solve uniquely, did not prevent the experimental technique that generated only scattering intensity from being adopted and used extensively. For scatteringin some cases, several different real-space models yield the same measured intensity pattern, so the uniqueness of the solution of the inverse problem is not guaranteed in that technique either. We believe that we have more than adequately demonstrated the significant potential and breadth of BCGE, making our advance suitable for publication in *Nature Communications*, and that many in several different fields will benefit by learning about this approach.

The introduction references GE used for analysis of biological macromolecules, i.e., nucleic acids and proteins in SDS, and gel-mobility shift assays for protein-DNA interactions. These interactions add complexity due to the lower electrophoretic mobility of the various species overall, uncertain charge differences, heterogeneity of interacting species and in complexes formed, overall size of the species and caging effects of the gel matrix, etc. While BCGE certainly could be applied to protein-DNA interactions in many cases, depending on the charge of the protein species, the advantages over standard gel-shift GE aren't immediately obvious.

> Our empirical demonstrations of BCGE are already more general than an assay intended only for biomolecules. Shift assays on biomolecules represent the closest prior

related work compared to our approach of BCGE, yet we have already pointed out some inherent limitations of EMSAs. The broad range of reaction types and revealing nature of the patterns formed that we have already demonstrated, particularly when simplified into the form of space-time plots, make the BCGE approach interesting and broadly applicable, irrespective of a particular potential future application of it involving biomolecules. While we have involved the biomolecule heparin in one reaction described in our original and in our revised submission, an entire future and more narrow BCGE study could be devoted simply to interactions of poly-nucleic acids, whether DNA or RNA, with proteins of various types. To facilitate such future work involving non-absorbing species, we have bolstered our Supplementary Discussion by describing an experimental extension of BCGE to biomolecules labeled with fluorescent tags and excited with UV light. This future work could address effects related to matrix type and concentration on the reactions and pattern generation as well as the range of proteins and poly-nucleic acids that could be assayed reasonably using BCGE. There will likely be some practical limitations (e.g. molecular mass, gel concentration) on the biomacromolecular interactions that could be assessed with BCGE. To us, this represents an intriguing frontier worthy of further specific studies based on the general idea of BCGE that we have introduced and demonstrated here. We have added 2-3 sentences to our discussion and concluding remarks suggesting that such studies would be interesting to perform.

In conclusion, this presents a very nice demonstration in principle of a clever approach that should motivate its further development. The additional work necessary to develop useful analytical techniques is significant.

> We thank Reviewer #3 for these largely positive comments, and we agree that further development is necessary to understand better how to interpret the complex patterns generated by a wide range of scenarios and reactions obtained using BCGE. While acknowledging that there is room for further future development of BCGE and the patterns generated by it, we nevertheless emphasize here that the breadth and range of our existing examples are quite strong and stand on their own. We think that timely publication of our localized reaction and detection methodology will attract the attention and interest of many researchers from a wide range of backgrounds, not just those who happen to be interested only in biomolecular interactions. We also think that our work will launch interesting scientific studies in a wide variety of directions, and collisions of biomolecules represent just one subset of these that can be studied in the future using our general BCGE approach. We again thank Reviewer #3 for his or her thoughtful comments, and we believe that our revised manuscript has been significantly improved through changes motivated by these comments.

REVIEWERS' COMMENTS:

Reviewer #1 (Remarks to the Author):

The authors have responded to all of my comments, and I am satisfied. I recommend publication.
